# Plant rarity in fire-prone dry sclerophyll communities

**DOI:** 10.1038/s41598-022-15927-8

**Published:** 2022-07-14

**Authors:** Meena S. Sritharan, Ben C. Scheele, Wade Blanchard, Claire N. Foster, Patricia A. Werner, David B. Lindenmayer

**Affiliations:** 1 Threatened Species Recovery Hub, Fenner School of Environment and Society, The Australian National University, Canberra, ACT 2601 Australia; 2 Fenner School of Environment and Society, The Australian National University, Canberra, ACT Australia

**Keywords:** Ecology, Environmental sciences

## Abstract

Understanding the responses of rare species to altered fire disturbance regimes is an ongoing challenge for ecologists. We asked: are there associations between fire regimes and plant rarity across different vegetation communities? We combined 62 years of fire history records with vegetation surveys of 86 sites across three different dry sclerophyll vegetation communities in Booderee National Park, south-east Australia to: (1) compare associations between species richness and rare species richness with fire regimes, (2) test whether fire regimes influence the proportion of rare species present in an assemblage, and (3) examine whether rare species are associated with particular fire response traits and life history. We also sought to determine if different rarity categorisations influence the associations between fire regimes and plant rarity. We categorised plant rarity using three standard definitions; species' abundance, species' distribution, and Rabinowitz's measure of rarity, which considers a species' abundance, distribution and habitat specificity. We found that total species richness was negatively associated with short fire intervals but positively associated with time since fire and fire frequency in woodland communities. Total species richness was also positively associated with short fire intervals in forest communities. However, rare species richness was not associated with fire when categorised via abundance or distribution. Using Rabinowitz's measure of rarity, the proportion of rare species present was negatively associated with fire frequency in forest communities but positively associated with fire frequency in woodland communities. We found that rare species classified by all three measures of rarity exhibited no difference in fire response traits and serotiny compared to species not classified as rare. Rare species based on abundance differed to species not classified as rare across each life history category, while species rare by distribution differed in preferences for seed storage location. Our findings suggest that species categorised as rare by Rabinowitz's definition of rarity are the most sensitive to the effects of fire regimes. Nevertheless, the paucity of responses observed between rare species with fire regimes in a fire-prone ecosystem suggests that other biotic drivers may play a greater role in influencing the rarity of a species in this system.

## Introduction

Rare species play important ecological roles that disproportionately contribute to the function and structure of species assemblages^1,2^. Rare species also face greater extinction risks than common species and are thus the focus of conservation efforts^3^. However, identifying the processes that drive the rarity of a species remain unclear. Rare plant species have diverse biological and life history characteristics^4,5^, suggesting that ecological processes may more strongly influence plant rarity than plant traits per se^6^. Disturbance is a major ecological process influencing the occurrence and abundance of species by acting as both a filter of the species pool and a driver of selection^7^. Understanding how rare species respond to disturbance is critical to informing the vulnerability of species to ecological disturbance and factors that that may drive their decline.

Several categorisations of rarity for above-ground species have been used to define what constitutes a rare species in ecology and conservation. Species rarity can be categorised using a species' local abundance, geographic range size, population density, or habitat breadth^3,5,8,9^. These different categorisations of rarity can capture various aspects associated with a species' response and vulnerability to environmental disturbance^8,10^. For instance, previous work defined rarity via the local abundance of a species^8^ to illustrate the effect of prescribed burns on the occurrence of rare and common species^11^. However, definitions of rarity based on more than one indicator of rarity, such as Rabinowitz's^9^ seven forms of rarity, can capture additional information associated with the possible causes of species rarity and their vulnerability to environmental disturbance^10^. For example, vertebrate species considered most rare by Rabinowitz's rarity classification were found to suffer the greatest decline in response to land-use changes^10^. However, current categorisations of rarity have yet to be used to clarify rare plant species' sensitivity to ecological disturbances.

Fires are important ecological disturbances that may be critical drivers of plant rarity^11,12^. Fire regimes often have unique impacts on vegetation communities^13,14^, shaping plant populations, community structure, and community dynamics^15–17^. Several studies have examined how fire regimes influence plant community composition and the abundance of certain species^11,13,14,18–20^. However, there has been limited work to examine how different fire regime characteristics can influence the presence and proportion of rare species across different vegetation communities. Attributes of fire regime characteristics that may shape plant rarity include fire frequency, time since fire, fire severity, and the length and variability of fire intervals (Supplemental Material S1, Table S1). Examining how total species richness, rare species richness (the number of different rare species present), and the proportion of rare species present in a community respond to fire regime characteristics may indicate possible drivers and extinction risks associated with plant rarity.

The functional traits of a species also may influence a species' rarity in relation to various aspects of a fire regime. Functional traits are related to ecosystem-specific survival and the performance of species^1^. For instance, short-lived fire ephemeral species may be rare in above ground vegetation in a system where recently burnt areas are rare. Conversely, slow-maturing fire-killed species that do not resprout after fire may become rare in areas subjected to frequent fires. Examining the fire response traits of species may offer a means to identify functional causes of species rarity and may therefore be useful in crafting species conservation strategies. However, there are limited studies comparing the traits of rare species and co-occuring common species in relation to fire disturbance^12,20,21^. We hypothesise that rare plants may have particular fire response traits and life history characteristics that influence their occurrence^12,13^.

This study explored whether three different categorisations of rarity, abundance, distribution and Rabinowitz's classification of a rare species influenced the associations observed between rare species and fire regimes. We combined 62 years of fire history records with vegetation surveys of 86 sites across three different vegetation communities in Booderee National Park, south-east Australia to: (1) compare associations between species richness and rare species richness with fire regimes, (2) test whether fire regimes influence the proportion of rare species present in an assemblage, and (3) examine whether rare species are associated with particular fire response traits and life history. We hypothesised that species classified as rare based on multiple characteristics (Rabinowitz's measure of rarity) would be more sensitive to the effects of fire regimes across different vegetation communities than species classified as rare based solely on abundance or distribution.

## Materials and methods

### Study area

We conducted this study in Booderee National Park (BNP), a 6500-ha reserve located on a coastal peninsula on the south coast of New South Wales, south eastern Australia (35°400 S, 150°400 E). The region has a temperate maritime climate and a long-term mean annual rainfall of 1212 mm over the last 20 years^22^. During the survey period, between September 2018 to February 2019, mean rainfall was 87.8 mm, the mean minimum temperature was 16 °C, and the mean maximum temperature was 23 °C. In the month prior to the vegetation sampling period (August 2018), the mean rainfall was 75.3 mm, the minimum temperature was 10 °C and the maximum temperature was 16 °C. The geology of the study area is dominated by Permian (260 million-year-old) undulating sandstone sequences overlain by quaternary sands at low elevations^23^, located on the southern edge of the Sydney Basin Geological system^24^. Soils in the region were derived from Permian sandstones and mudstones, talus derived from the sandstones, valley-fill alluvium ranging from coarse sandy material to flood-plain muds^25^.

Booderee National Park is included within the floristically diverse Sydney Coastal Heath and Sydney Coastal Dry Sclerophyll Forest vegetation communities^26^. The Park is dominated by dry sclerophyll vegetation consisting primarily of forest (45.1% of BNP), heath (15.3%) and woodland (12.9%) (Taws 1997); the three most widespread vegetation communities in BNP. Each community is distinguished by the height, cover, and the identity of species in the canopy. The distribution of the different vegetation communities depends primarily on abiotic factors^26,27^. For more detailed descriptions of the three vegetation communities, see Supplemental Material S2.

### Field surveys

Our study was conducted across 86 sites, a subset of 110 long-term monitoring sites established in 2003 across Booderee National Park (BNP) to study biodiversity responses to fire. These sites were established using a stratified randomised approach, where sites were selected so that they were stratified by vegetation type and time since fire at the time of site selection in 2003. The sites were distributed widely throughout the park, ensuring the number of sites per vegetation community was selected proportional to the area that vegetation community covered. For a detailed description on the selection of the study sites selected, see Lindenmayer et al.^28^.

Our 86 sites covered the three major vegetation communities in BNP: forest (39 sites), woodland (22 sites), and heath (25 sites). In each site, we established a 10-m by 10-m square plot and surveyed 100 one-metre by one-metre quadrats, creating a 10 by 10-unit grid. We identified each vascular plant species rooted within each one-metre square quadrat. The total abundance of a species at a site was the number of one-metre quadrats in which a species was recorded. The presence of vegetatively spreading species also was recorded within each one-metre square quadrat. If a clone of the species occupied more than one quadrat, we counted it in all quadrats present. For species with large wooden stems such as *Eucalyptus* and *Xanthorrhoea* species that occupied more than one quadrat, we recorded their presence in the quadrat in which they occupied the greatest area.

We conducted plant surveys from September 2018 to February 2019, which included the prime flowering season (September – November 2018). We attempted to identify each plant to species level. However, species that could not be identified to this level were given a unique identifier. We acknowledge that there is an unlikely possibility that certain unidentified species may be an unidentified juvenile form of an identified species. As we were unable to distinguish between these two scenarios, we included all unidentified species present in our data analyses.

### Fire in Booderee National Park

Records of fire history, fire perimeters and causes of fire (wildfire or prescribed fire) have been well-documented since 1957 for BNP. Using these data, we calculated the time since fire and fire frequency occurring at each of the 86 sites in which we conducted vegetation surveys. We also recorded if each site had an inter-fire interval of less than seven years, as a binary variable. The seven-year threshold for the short fire interval was made according to Bradstock & Kenny^29^ where a site experiencing an inter-fire interval of less than seven years is outside the recommended domain of ‘acceptable’ fire intervals (seven to 30 years) for dry sclerophyll heaths and woodlands. The occurrence of a short fire interval is known to influence the dominance and abundance of species post-fire (Supplemental Material S1, Table S1). Summary data of the fire regime variables for each vegetation community are provided in Supplemental Material S2.

### Plant rarity classification

We focused our study only on species that were present in above-ground vegetation. We did not consider species present in the soil seed bank. Above-ground vegetation is dissimilar to species in the seed bank due to the differing effects of disturbance above ground and belowground^30^. We calculated overall species abundance by averaging the site abundance scores (the number of quadrats occupied within a site) of each species across all sites and all vegetation communities. The distribution of a given species was defined as the number of sites in which a species was found.

There are multiple ways to classify plant rarity; we used the three most common categorisations of rarity found in the literature. Using three categorisations of rarity allowed us to explore whether categorisation criteria influenced observed patterns. For the complete list of species and their rarity classifications for each rarity categorisation, see Supplemental Material S3. The three categorisations of rarity we used were:

1. *Rarity by Abundance, according to Gaston*^8^*.* Rare species were identified as those whose abundance scores fell below the 25th quantile of average species abundance across the total number of sites surveyed. Those above the 25th quantile were classified as common.
2. *Rarity by Distribution, according to Gaston*^8^. Rare species were identified as those found in the lowest the 25th quantile for the number of sites a species was found across the total number of sites surveyed.
3. *Rabinowitz's classification of a rare species*^9^. In our study, species classified as most rare were those classified according to Rabinowitz's definition. Here, a rare species has a small local population size, a narrow distribution, and high habitat specificity (categorised as NSS; Table 1). A species with a small local population had an abundance that was less than 50% of the abundance of the most abundant species in our dataset. In accordance with Rabinowitz’s binary split of a narrow or wide distribution as an indicator of geographical rarity, a species present in less than 43 of the 86 sites surveyed was classified as having a narrow distribution. The species also had high habitat specificity (found in only one vegetation community). We examined only species classified as most rare by Rabinowitz (Table 1; NSS) in this study. Species assigned to the other Rabinowitz rarity categories were classified as 'not rare' for analysis.

**Table 1:** Rabinowitz's (1981) categorical classification of the seven forms of rarity for species, based on species abundance, distribution and habitat specificity.

Rarity category	Descriptions according to Rabinowitz			Definitions applied to the study area		
	Distribution	Population size	Habitat specificity	Distribution (number of sites)	Total abundance across all sites (46 occurrences)	Habitat specificity
WLU	Wide geographic distribution	Somewhere large	Unspecific	> 43 sites	> 46	3
WSU	Wide geographic distribution	Everywhere small	Unspecific	> 43 sites	< 46	3
WLS	Wide geographic distribution	Somewhere large	Specific	> 43 sites	> 46	1
WSS	Wide geographic distribution	Everywhere small	Specific	> 43 sites	< 46	1
NLU	Narrow geographic distribution	Somewhere large	Unspecific	< 43 sites	> 46	3
NSU	Narrow geographic distribution	Everywhere small	Unspecific	< 43 sites	< 46	3
NLS	Narrow geographic distribution	Somewhere large	Specific	< 43 sites	> 46	1
NSS (rare species)	Narrow geographic distribution	Everywhere small	Specific	< 43 sites	< 46	1

Within Booderee National Park, a total of 86 sites were surveyed, and species abundance ranged from one to 92 occurrences within a site. The number of habitats indicates how many vegetation communities out of the three different vegetation communities surveyed the species was present in.

### Data analysis

#### Associations between fire history, vegetation community, species richness, and rare species richness

We examined associations between fire history and vegetation community with species richness and rare species richness at a site. Species richness was defined as the total number of species detected within the 100 m^2^ quadrat at each of the 86 sites surveyed. Of the 455 presumed species found, 65 species were not identifiable (14.3%). We included all 455 species for analyses examining the associations between fire regimes, vegetation community, species richness, and rare species richness.

We conducted a variance inflation factor analysis to check for multicollinearity^31^ between fire regime variables across all 86 sites to be used in the full model using the "car" package in R^32^. We found a strong correlation (> 0.7) between the total number of fires (wildfires and prescribed fires) at a site and the number of wildfires at a site. We found that 68% of all plot-level burns that occurred were wildfires, while the remaining 32% of fires that occurred were either hazard reduction burns, controlled burns, hazard reduction burns that became wildfires or were not classified. Consequently, we did not distinguish between wildfires and prescribed fires in our analyses. We created a matrix of correlations among the fire regime variables for analyses across all sites and each vegetation community (see Supplemental Material S2, Tables S1 and S2).

We fit negative binomial generalised linear models using the "glm.nb" function in the "MASS" package^33^. We first examined if site species richness was associated with fire regimes. Species richness was the response variable and the predictors in the full model were: vegetation community * (log(time since fire) + fire frequency + short interval) + northing (cos (aspect)) + easting(sin(aspect)) + elevation. We tested the full model with fire frequency as a linear variable and as a polynomial variable to detect the possibility of non-linear associations between our response variable and our predictor variables. We compared the linear and non-linear models via their AIC and selected the model that had the most parsimonious fit (Supplemental Material S2). We log-transformed time since fire as the effects of time since fire are expected to be non-linear, where a one year difference in time since fire would be expected to be more influential in the first few years post-fire than at 40 years or more post-fire^34^. We included the topographic variables aspect and elevation as covariates in the models to reduce potential confounding fire effects with spatially related environmental factors. Elevation was the height above sea level in metres. We split aspect into two linear components. The easting component was the sine of the prevailing aspect of the site. The northing component was the cosine of the prevailing aspect of the site. We used AIC corrected for small sample sizes (AICc) to rank subsets of the full model using the "dredge" function in the "MUMIn" package^35^ and determined the most strongly supported model for species richness. We tested all datapoints for high leverage using the "hatvalues" function for the most strongly reported model. We inspected residual diagnostics for the model using the *"DHARMa"* package^36^.

We then examined whether the number of rare species at a site (rare species richness; the number of rare species detected within each 100 m^2^ quadrat) responded differently to species richness. We followed the same procedure used for species richness but changed the response variable to rare species richness at a site. We repeated this procedure for all three categorisations of rarity. For all model outputs containing the model estimates, standardised regression coefficients and estimated 95% confidence intervals, see Supplemental Material S4.

#### Associations between the proportions of rare species in an assemblage with fire history and vegetation community

We fit a generalised linear model with a binomial distribution to investigate whether differences in fire history and vegetation community influenced the proportion of rare species relative to total species richness at a site. The proportion of rare species at a site was our response variable. The full model was: vegetation community * (log(time since fire) + fire frequency + short interval) + northing (cos (aspect)) + easting(sin(aspect)) + elevation. We tested the full model with fire frequency as a linear variable and as a polynomial variable to detect the possibility of non-linear associations between our response variable and our predictor variables. We used AIC corrected for small sample sizes (AICc) to rank subsets of the full model using the "dredge" function in the "MUMIn" package^35^. For all model outputs containing the model estimates, standardised regression coefficients and estimated 95% confidence intervals, see Supplemental Material S5.

### Traits of rare species

We examined if rare species were associated with particular fire response traits, seed storage location, serotiny, and life history compared with species not classified as rare. These traits were collated for all plants identified to species level from the AusTraits database^37^. For the full list of species and their associated traits, see Supplemental Material S2, Table S1).

We first conducted a Fisher's exact test to determine if rare species had different fire response traits to species not classified as rare species. We then conducted a Fisher's exact test to determine if rare species had different seed storage locations compared to species not classified as rare. Next, we conducted a Fisher’s exact test to determine if serotiny differed between species classified as rare and not rare. Finally, we conducted a Fisher's exact test to observe if life history differed between species classified as rare and not rare for all three categorisations of rarity. For further information on the proportion of species attributed with each trait data per vegetation type and form of rarity, see Supplemental Material S6.

We conducted all data analyses in R v 4.0.2 (R Core Team 2020). Our significance threshold was P < 0.05 for all analyses.

## Results

### Species richness and rare species richness responses to fire regimes characteristics

**Figure 1:**
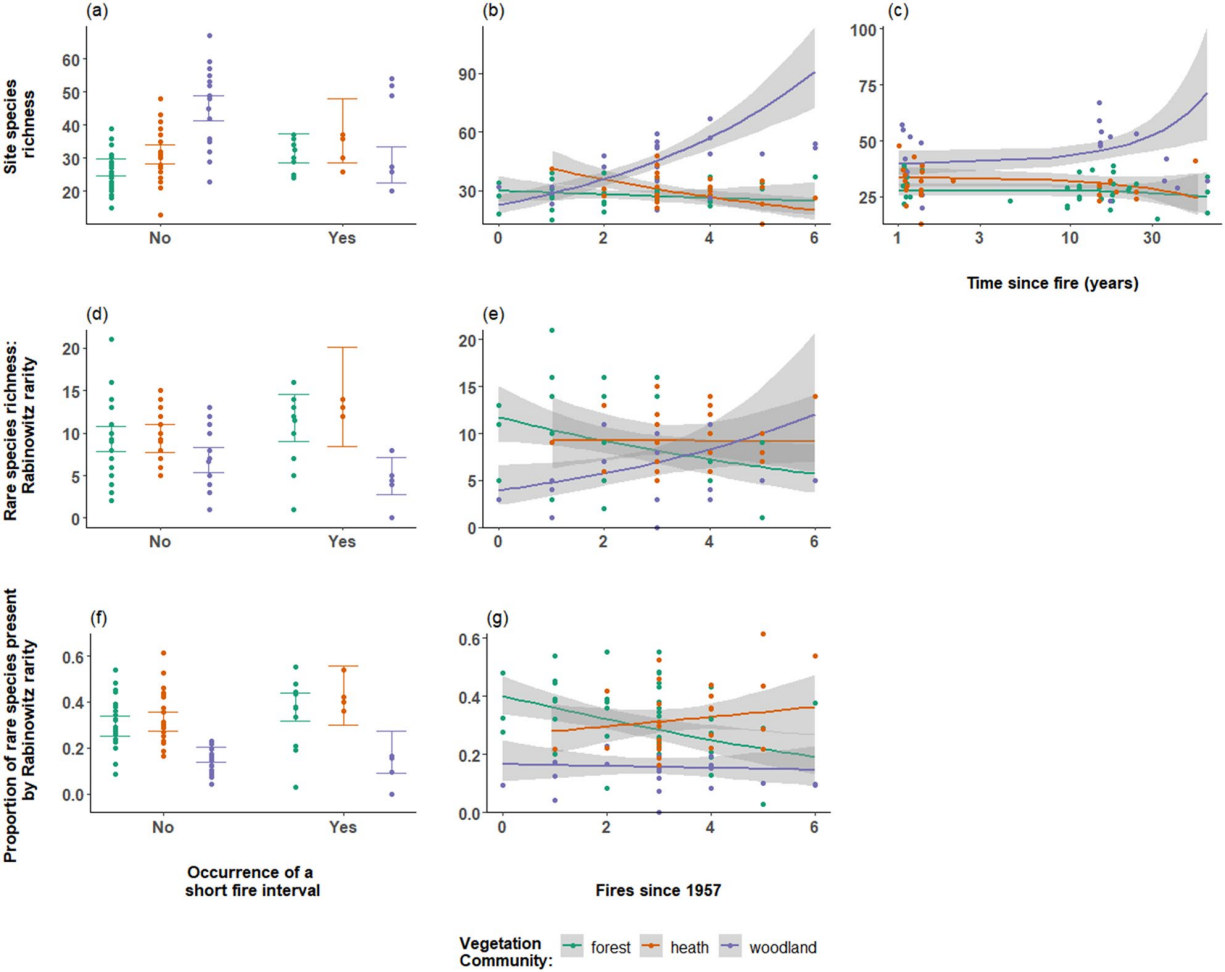
Associations between the occurrence of a short interval, the number of fires since 1957 (fire frequency) and time since fire, with site species richness (**a**–**c**); Rabinowitz rare species richness with the occurrence of a short interval and the number of fires since 1957 (**d**,**e**); and the proportion of Rabinowitz rare species present (**f**,**g**) across the three major vegetation communities in Booderee National Park; forest (green), heath (orange), and woodland (purple). Predicted values are from the top-ranked model and 95% prediction confidence intervals are shown. Closed circles show raw site vegetation data for each vegetation community. For time since fire (**c**), the log of time since fire was used for analyses but the predicted values are shown on the original scale of time since fire in years.

The most strongly supported reported model for species richness included fire frequency, time since fire and the occurrence of a short fire interval (< 7 years) interacting with vegetation type, indicating that the associations between the three fire variables and species richness differed among heath, forest, and woodland communities. We found species richness was significantly negatively associated with the occurrence of a short fire interval in woodland communities (P < 0.001, Fig. 1a.). Species richness was significantly positively associated with fire frequency and time since fire in woodland communities (P < 0.001 Fig. 1b.; P < 0.001, Fig. 1c.). The occurrence of a short fire interval was positively associated with the species richness in forest sites (P = 0.045, Fig. 1a). We found no significant associations between species richness and the fire regime variables in heath communities.

The most strongly reported model for rare species richness by abundance and distribution included only vegetation type. Rare species classified by their abundance and distribution showed no associations between rare species richness and the fire regime variables examined. With Rabinowitz's measure of rarity, the most strongly supported model included fire frequency and the occurrence of a short fire interval interacting with vegetation type. We found that in woodland communities, rare species richness was significantly negatively associated with the occurrence of a short fire interval when species were classified with Rabinowitz's measure of rarity (P = 0.001, Fig. 1d), but positively associated with fire frequency (P = 0.001, Fig. 1e). In forest communities, Rabinowitz rare species richness was positively associated with the occurrence of a short fire interval (P = 0.010, Fig. 1d), but was negatively associated with fire frequency (P = 0.017, Fig. 1e). Heath communities showed no associations between rare species richness and all fire regime variables examined.

### The proportion of rare species present in response to fire regimes

With abundance as a measure of rarity, the most strongly supported model included only vegetation type. With distribution as a measure of rarity, the top-ranked model included time since fire (logged) and vegetation type. However, no associations were present between the proportion of rare species present with time since fire across all three vegetation communities with the two measures of rarity.

Using Rabinowitz's measure of rarity, the most strongly supported model included the occurrence of a short fire interval, and fire frequency interacting with vegetation type. We found that the proportion of rare species present was significantly positively associated with the occurrence of a short fire interval in forest communities (P = 0.045, Fig. 1f). We found the proportion of rare species was significantly negatively associated with fire frequency in forest communities (P = 0.004, Fig. 1g). Rabinowitz rare species in heath communities were positively associated with fire frequency (P = 0.006, Fig. 1g).

### Traits of rare species

Across all three rarity categorisations, the dominant fire response trait of species classified as rare were resprouters (abundance; 72%, distribution 66%; Rabinowitz; 66%). For all three rarity categorisations, there were only eight serotinous rare species when rare species were identified by abundance (88%), 14 by distribution (100%), and 26 by Rabinowitz’s rare species criteria (87%). Rare species, across all three measures of rarity stored seeds in only soil seedbank rather than in the canopy or were able to store seeds in both the soil and canopy (abundance; 73%, distribution 88%; Rabinowitz; 81%). Rare species were also most frequently perennial species (abundance; 90%, distribution 94%; Rabinowitz; 96%). Based on a Fisher's exact test, we found that rare species classified by all three measures of rarity exhibited no difference in fire response traits and serotiny compared to species classified as not rare (P > 0.05). We found that rare species categorised only by abundance differed across each life history category (P = 0.014), where most rare and not rare species were perennial but one rare species was biennial. Rare species categorised by their distribution differed to species classified as not rare in their seed storage location (P = 0.003), where most rare species stored seed in the soil, but species classified as not rare stored seed in both the canopy and the soil. For tables containing the number of rare and not rare species under each category for fire response traits, serotiny, seed storage location, and life history, see Supplemental Material S6.

## Discussion

Our results show that species richness and rare species richness respond to fire regimes differently across woodland, forest and heath communities. Rare species did not respond to fire regime variables when categorised by abundance or distribution. However, with Rabinowitz's measure of rarity, the proportion of rare species was negatively associated with fire frequency in forest communities but positively associated with fire frequency in heath communities. Rare species also did not differ in their fire response traits and serotiny to species categorised as not rare across all three measures of rarity. We hypothesise that these differences in fire history associations between species richness and rare species are due to the differences in abiotic and biotic conditions across each vegetation community.

### Species richness and rare species richness responses to fire regime characteristics

Species richness and rare species richness showed similar associations with fire frequency and a short fire interval in woodland communities. This finding suggests that rare species account for a large proportion of total species richness in woodland communities (Supplemental Material S4, Table S2) and fire regimes play a key role in influencing species composition. However, total and rare species richness did not respond to fire regimes in heath communities across all three categorisations of rarity, while in forest communities, only total species richness responded to the occurrence of a short interval. The differences in the responses of total species richness and rare species richness with fire across each vegetation community suggests that differences in environmental and competitive conditions of each vegetation community may play a key role in influencing the presence of rare species^38–40^. In most ecosystems, rare species are often assumed to account for the bulk of species richness and can be indicative of environmental change occurring within an ecosystem^41–43^. Rare species can promote the delivery of multiple ecosystem functions such as improving resistance to invasions^44^, providing multiple indirect interactions and unique functional roles^1,45^. However, common and dominant species can control ecosystem function and may offset the negative effects of species loss by maintaining above-ground net primary productivity^46^. Since the number of rare species present at a site in heath communities was not associated with fire, it may be that common species are more responsible for changes in species richness patterns in this vegetation type^47^. Additionally, environmental processes other than recent fire history may be driving variation in species richness in heath communities. Examining changes in the composition of common species and other environmental processes such as herbivory^48^ may be a critical future step in determining what conservation management strategies are best for conserving ecosystem functionality. For instance, strategies for conserving vegetation in heath communities may need to focus on the changes in common species rather than the richness of rare species in our study system. However, in woodland communities, rare species richness behaved similarly to species richness, suggesting that conservation efforts should focus on understanding rare species composition and how rare species contribute to ecosystem function.

### The proportion of rare species present in response to fire regimes

We found that the proportion of rare species present based on Rabinowitz’s classification at forest sites was negatively associated with frequent fire. Most of the rare species in our study system were resprouters. Frequent fires and short intervals between fires may give resprouting rare species a temporary fitness advantage over non-resprouters after fire^49^. Our trait analyses demonstrate that the fire response traits and life history of species were mostly not associated with rarity, indicating that the post-fire evolutionary strategies of species are not confounded with the temporal rarity of a species in response to fire. As most rare species were resprouters in our system, our findings indicate that other additional post-fire interactions may influence the rarity of species in forest communities. For example, previous work in this system found that the interaction between herbivory and fire in forest systems can lead to a decline of palatable plants^48^, and dominance by bracken (*Pteridium esculentum),* a common plant in forest communities that can regenerate rapidly following fire^50^. The presence of macropod grazers in conjunction with rapid bracken regrowth may lead to reduced abundance of palatable plants, and hence the lower proportion of Rabinowitz rare species in response to frequent fires, suggesting other biotic interactions such as herbivory may be a limiting factor for early successional rare species^51^.

The difference in vegetation structure between these communities also suggests that competition from overstory species could slow the growth of rare species in forests and woodlands in response to fire. Woodlands and forests support a canopy of *Eucalyptus* species compared to heath communities with dense thickets of *Banksia* species^24^. Our study found a higher proportion of rare species present in heath communities with frequent fire. Heath communities with a higher fire frequency may have led to a decrease in competition and cover from the thickets of common *Banksia* species, providing new conditions suitable for rare species to survive and persist post-fire. Rare species richness in heath communities may not be responding directly to fire regimes but to the changes in environmental conditions and resources post-fire. For instance, heath communities tend to be in shallow soils and in areas of low productivity^26,52^. Recurrent fires can reduce available soil resources^53^. Fires can also temporarily mobilise resources, that were previously locked up in vegetation^54^. A higher fire frequency in heath communities of BNP may also reduce the abundance of dominant species and prevent the formation of dense *Banksia* thickets, consequently providing more available space and greater canopy openness that allow rare resprouting species to persist. Thus, rare species may take advantage of the change in resources and reduction in competition of space and light from *Banksia* species and other dominant species. Future work should thus focus on the interactive effects of fire and plant interactions between rare and common plant species in understanding how rare species respond to fire disturbance across time.

### Traits of rare species

We found little evidence that species categorised as rare have particular life history or fire response traits compared to species not categorised as rare. The lack of fire response traits associated with rare species indicates that the associations found between fire and rare plants in this study could not be explained by differences in evolved fire responses between rare and not rare plants. Despite some studies showing that contemporary fire regimes are a threat to a large number of plant species and communities, there are few empirical studies examining how rare species are associated with fire regimes^11,20,55–57^. One explanation for this is that fire is influencing community dynamics and composition by altering resource availability, competitive interactions, or herbivore interactions^58,59^, rather than direct interactions with species’ vital attributes. Previous work elsewhere has found that fire regimes may be associated with rarity but these have been alongside other environmental attributes such as rainfall^11,56^ and herbivory^51^. A trait-based approach to identify the functional causes of rarity in a fire-dependent ecosystem in the North Carolina's Sandhills region found only three of 18 functional traits were associated with fire suppression. Thus, our findings suggest that in fire-prone ecosystems, a species' fire response traits may not be good predictors of plant rarity.

### Different measures of rarity

Finally, our analyses revealed that using abundance or distribution as a measure of rarity provided little insight into patterns of responses of rare species to fire regimes in comparison to Rabinowitz's measure of rarity. Comparing different rarity categorisations in response to the same fire regime variable demonstrates that assessing species vulnerability to changes in the environment should consider multiple aspects of rarity. Abundance or distribution alone may not be a suitable measure of plant rarity at a fixed point in time, particularly in fire-prone ecosystems. Rarity definitions that consider abundance, distribution and habitat specialism can provide greater detail in understanding a species' sensitivity to both natural and anthropogenic changes in a landscape^10^. However, future work seeking to examine the responses of rare species to fire regimes requires expanding the concept of rarity to include temporal dynamics in addition to Rabinowitz's three attributes of rarity (abundance, distribution and habitat specialism). Temporal rarity can indicate whether a species is persistently rare or transiently rare in relation to environmental change across time^55^. Considering temporal rarity in future work can provide more information about the vulnerability of a species to decline and possible extinction risk^10,55^.

Various definitions of rarity can demonstrate the different threats rare species face locally versus globally^3,10^. Our study, which used the most commonly used methods for classifying rare species recognises that rarity classifications are scale dependent. These rarity measures can also result in situations where some species that have large geographic distributions but are locally scarce are classified as rare^60–62^. Most important for application of our study, however, is the fact that when it comes to conservation decisions and efforts, the local scale is most often of interest. Our study provides an example of how the responses of rare species to disturbance is highly context-dependent^5^, and can be dependent on the definition of rarity used. The results indicate that understanding the response of rare species requires considering the vegetation community context of a species. Thus, conservation efforts concerning rare species need to be couched in local terms and require focused efforts, especially as fire regimes can have very different effects on different vegetation communities^13,14^.

### Caveats

Our study examined above ground vegetation only in response to fire regimes influencing rarity. Understanding species richness and the proportion of rare species present above ground can provide vital information on ecosystem functioning and resilience^2^. We did not consider species present below-ground in the soil bank. The species composition of standing vegetation can differ greatly to species present in the soil seed bank^63^. Plants that are rare or absent in standing vegetation communities may be abundant in the soil seed bank. Their abundance can be influenced by seasonal dormancy cycling and sporadic events such as a high severity fire and consecutively high rainfall years^16,64^. Consequently, rare species in the soil bank may respond differently to fire than rare species in standing vegetation. Attempting to understand the responses of rare species below-ground compared to standing vegetation with fire regimes is an avenue for future work in examining rare species vulnerability to extinction.

A mechanistic explanation of the demonstrated relationships between fire regimes, rare species and total species richness with available plant functional traits was not possible in this study. That was due to insufficient sample sizes for rare species across our study area that would be required for such an exploration. Future work, requiring large datasets of rare species occurrence and their functional traits in relation to fire regimes via long term monitoring studies can assist in detecting the effects of altered disturbance regimes on rare species occurrence patterns and assist in forecasting future changes in rare species abundance and occurrence.

## Conclusion

The patterns we observed indicate that the responses of rare plant species to environmental disturbance are not homogenous. Instead, associations between fire regimes with the presence and proportion of rare species present are unique to different vegetation communities. Our study suggests that Rabinowitz's measure of rarity may provide more detail relative to other approaches in detecting the responses of rare species to various aspects of a fire regime. Rabinowitz rare species responded to fire frequency and the occurrence of a short fire interval which we did not detect by other commonly used categorisations of rarity. These responses of rare species to fire regime variables could not be associated with differences in fire response or life history traits between rare and not rare species. Our results suggest that future studies seeking to understand the drivers of rarity in fire-prone ecosystems should consider how biotic influences such as plant competition and herbivory influence rarity.

## Supplementary Information

Supplementary Information.

## Data Availability

Data is available from the Dryad Digital Repository: 10.5061/dryad.cc2fqz68p.
