# Supplementary material for: Plant rarity in fire-prone dry sclerophyll communities
Source: Sci Rep. 2022 Jul 14;12:12055. doi: 10.1038/s41598-022-15927-8 (PMC9283327; doi:10.1038/s41598-022-15927-8)
Supplement: Supplementary file 1 — Supplementary Information. [file 41598_2022_15927_MOESM1_ESM.docx]

# Plant rarity in fire-prone dry sclerophyll communities - Electronic Supplemental Material

Meena S. Sritharan, Wade Blanchard, Claire N. Foster, Patricia A. Werner, David B. Lindenmayer^,^

# Electronic Supplemental Material 1

Table 1. Summary of certain fire regime characteristics and their influence on plant traits, species richness, and abundance of species identified from the literature.

| Fire regime variable | Effects of fire regime variable on: | | |
| --- | --- | --- | --- |
|  | Traits | Species richness | Plant abundance |
| Fire frequency | High fire frequency can influence the survival of species in relation to height ^1^, growth form, and age till reproductive maturity ^2^.  Species with the capacity to resprout after fire were considered at risk of local extinction under regimes of frequent fire ^3^.  Resprouters were able to cope with more frequent fire than non‐sprouters in scrub heath ^4^ | Positively associated with species richness in woodland communities ^5^  High fire frequency can influence species richness negatively in heath and reduce populations of heath species ^5,6^.  Annual fire reduced species richness and diversity, particularly in the absense of grazing in native tallgrass prairie ^7^.  Sites exposed to high‐frequency fire contained the fewest number of species, whereas sites exposed to a low frequency of fire were characterised by the greatest shrub species richness in C4-dominated grassland ^8^. | Intermediate fire frequency increased the abundance of the common shrub *Cornus drummondii* relative to a low fire frequency ^9.^.  Frequent fire can deplete populations of heathland species with the dominant shrub species *Banksia ericifolia* being most affected ^10^. |
| Time since fire | Associated with relative plant cover and associations differed across plant groups ^11^.  Resprouting species increased and obligate seeders decreased along a fire–productivity gradient ^12^.  Seed production varies in relation to the age and size of the plants and time since fire ^13^. | Decrease in species richness with increased time since fire in dry open forests ^14^ ^n^, heath ^15^, and low open woodlands ^p^.  In long unburnt Mallee-heath, increasing time since fire was associated with a decline in species density; species senescence occurred ^16^. | Had a dominant influence on the abundances of species in healthy Eucalyptus woodland ^17^.  With increasing time since fire:   - large shrubs increased in abundance while herbs and small shrubs decreased in abundance in dry sclerophyll vegetation ^18^. - the abundance of shrubs, grasses, ephemeral and twining perennial forms increased one year after fire but declined with time since fire ^18^. - mallee communities increased in stature, 55 years post-fire ^19^. - ephemeral grasses, forbs and *Triodia spp*.seedlings decreased in abundance in sand-ridge plant communities ^20.^. |
| Short fire intervals | A reduction in the number of fire-sensitive species in dry sclerophyll vegetation, particularly common shrub species with a canopy-stored seed bank and non-leguminous species with a soil stored seed bank ^21.^.  Short fire intervals can have negative effects on resprouting vegetation^3,21,22^. | A decrease in plant species richness immediately after a fire in Mediterranean forests sites with a short interval between fires ^11^.  A reduction in the number of species present; a decrease in the evenness of fire‐sensitive species ^10^.  Increasing variability of the length of the inter‐fire intervals is associated with an increase in the species richness of both fire‐sensitive and fire‐tolerant species in dry sclerophyll vegetation ^21^. | Reduced time available for dominant species to re‐establish before the next fire ^23^.  Shorter fire intervals are associated with a reduction in the number of species present in dry sclerophyll vegetation ^21^. |

# Electronic Supplemental Material 2

**Vegetation community and fire history information for Booderee National Park**
Booderee National Park (BNP) is a ~6,500-ha reserve located on a coastal peninsula on the south coast of New South Wales, south-eastern Australia (35°400 S, 150°400 E). The region has a temperate maritime climate and an average yearly rainfall of 1212 mm over the last 20 years ^27^. Booderee National Park falls under the floristically diverse Sydney Coastal Heath and Sydney Coastal Dry Sclerophyll Forest vegetation communities ^28^. The park is dominated by dry sclerophyll vegetation consisting primarily of forest (45.1% of BNP) followed by heath (15.3%) and woodland (12.9%) vegetation communities ^29^.

The forest vegetation community (trees have touching crowns, >20m tall ^29^) is the most extensive vegetation community across BNP, dominated by *Eucalyptus pilularis*, *Corymbia gummifera*, and *Eucalyptus botryoides*. The mid-storey comprises of *Banksia serrata, Acacia longifolia*, and *Monotoca eliptica*, while the understory is dominated by *Pteridium esculentum* and *Lomandra longifolia*.

Heathlands are treeless and are instead dominated by a variety of shrub species usually less than two metres tall with small narrow leaves forming part of the canopy^29^. Dry heath communities are dominated by *Banksia ericifolia, Allocasuarina distyla, Sprengelia incarnata, Baeckea imbricata, Isopogon anemonifolius and Hakea teretifolia,* while in wet heath *Gahnia clarkei*, *Gleichenia dicarpa*, *Leptospermum* or *Melaleuca* species can also be found.

Woodland communities (trees have separated crowns, <20m tall ^29^) have a high variation in crown cover from closely spaced to widely separated, dissimilar from forests based on lower stature and species composition. The overstorey is comprised of *Eucalyptus sclerophylla*, *C. gummifera*, *and B. serrata*; the mid-storey is dominated by *B.serrata* and *C.gummifera*, and the understory is composed of *P. esculentum*, *B.serrata*, *Lambertia formosa*, *Acacia longifolia*, *Acacia suaveolens*, and *L. longifolia*. Woodland vegetation communities occur in transitional areas between forests and heath^29^. For further details on the description of vegetation communities present in BNP see Taws ^29^.


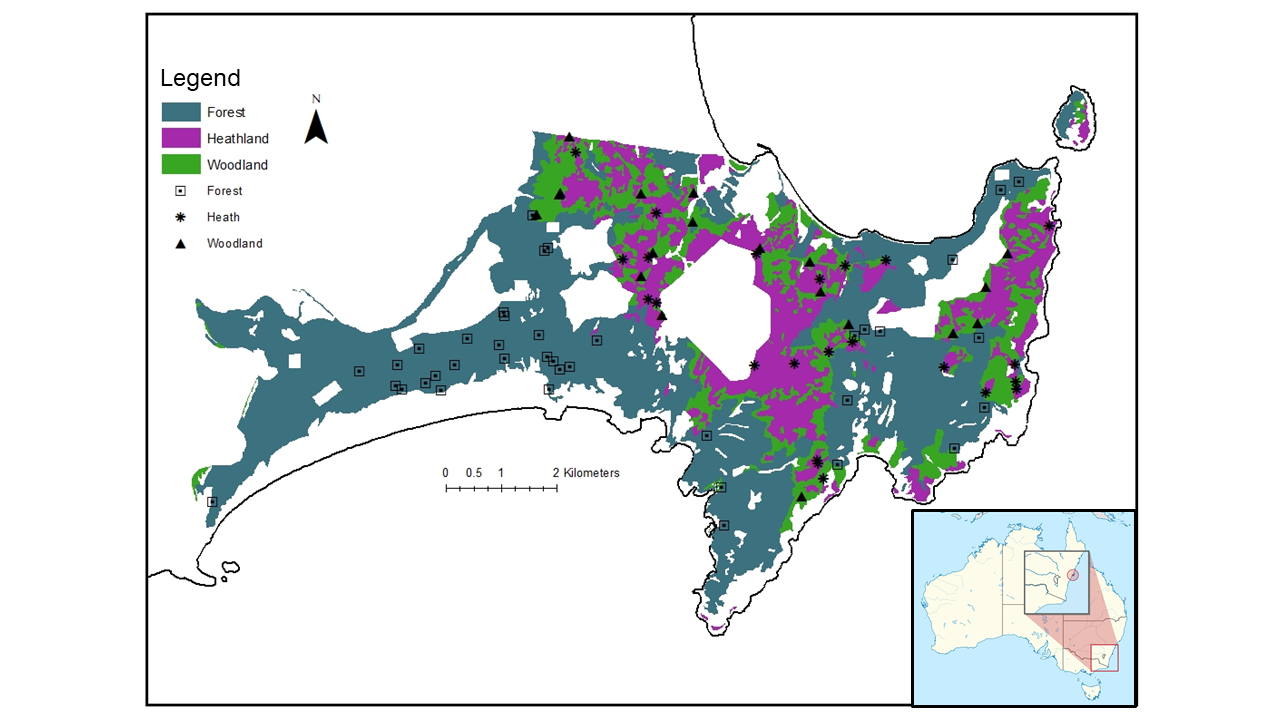


Figure 1. Map of Booderee National Park, the distribution of the three most dominant vegetation communities and the 86 sites surveyed. Map was created using ArcGIS® software by Esri. ArcGIS® and ArcMap™ are the intellectual property of Esri and are used herein under license. Copyright © Esri. All rights reserved.

Table 1. Summary of descriptive data of fire frequency, time since fire and number of short intervals for each vegetation community.

| **Fire regime variables** | **Forest** | **Heath** | **Woodland** |
| --- | --- | --- | --- |
| Minimum fire frequency | 0.00 | 1.00 | 0.00 |
| Maximum fire frequency | 6.00 | 6.00 | 6.00 |
| Mean fire frequency | 2.53 | 3.52 | 3.09 |
| Median fire frequency | 3.00 | 3.00 | 3.00 |
| Minimum time since fire | 1.06 | 1.01 | 1.04 |
| Maximum time since fire | 62.80 | 53.63 | 62.80 |
| Mean time since fire | 14.64 | 8.70 | 14.64 |
| Median time since fire | 11.27 | 1.36 | 14.79 |
| Number of short intervals across all sites | 11 | 3 | 5 |

Table 2. A matrix of the correlations among the fire regime variables used for analyses across all sites used for analysis

|  | Fire frequency | The occurrence of a short fire interval | Log (time since fire, years) |
| --- | --- | --- | --- |
| Fire frequency | 1.000 | 0.406 | -0.625 |
| The occurrence of a short interval | 0.403 | 1.000 | -0.043 |
| Time since fire (years - logged) | -0.625 | -0.043 | 1.000 |

Table 3. A matrix of the correlations among the fire regime variables used for analyses across forest, woodland and heath vegetation communities.

| **Vegetation community** | **Fire regime variable** | **Fire frequency** | **The occurrence of a short interval** | **Time since fire (years) -logged** |
| --- | --- | --- | --- | --- |
| **Forest** | Fire frequency | 1.000 | 0.481 | -0.742 |
|  | The occurrence of a short interval | 0.481 | 1.000 | -0.069 |
|  | Time since fire (years) | -0.742 | -0.069 | 1.000 |
| **Woodland** | Fire frequency | 1.000 | 0.544 | -0.550 |
|  | The occurrence of a short interval | 0.544 | 1.000 | -0.195 |
|  | Time since fire (years) | -0.550 | -0.195 | 1.000 |
| **Heath** | Fire frequency | 1.000 | 0.385 | -0.453 |
|  | The occurrence of a short interval | 0.385 | 1.000 | 0.032 |
|  | Time since fire (years) | -0.453 | -0.032 | 1.000 |

Table 4. Comparison of linear and non-linear models tested for species richness and rare species richness

| Model | AIC | Df |
| --- | --- | --- |
| Species richness – non-linear | 593.29 | 22 |
| Species richness - linear | 584.56 | 16 |
| Abundance – non-linear | 559.68 | 22 |
| Abundance - linear | 355.75 | 16 |
| Distribution – non-linear | 388.36 | 22 |
| Distribution- linear | 383.45 | 16 |
| Rabinowitz – non-linear | 485.01 | 22 |
| Rabinowitz - linear | 479.09 | 16 |

Table 5. Comparison of linear and non-linear models tested for the proportion of rare species present at a site

| Model | AIC | Df |
| --- | --- | --- |
| Abundance – non-linear | 334.66 | 18 |
| Abundance - linear | 334.23 | 15 |
| Distribution – non-linear | 361.21 | 18 |
| Distribution- linear | 360.08 | 15 |
| Rabinowitz – non-linear | 439.6153 | 18 |
| Rabinowitz - linear | 437.0637 | 15 |

# Electronic Supplemental Material 3

Plant list for all the identified species for which traits could be obtained found across the 86 sites surveyed and their classification of rarity according to Gaston’s ^30^ rarity by abundance, in which a species that falls below the 25^th^ quantile for average abundance across the sites was classified as rare; Gaston’s rarity by distribution, in which a species that falls below the 25^th^ quantile for the distribution of species across all sites across the park and Rabinowitz’s ^31^ seven forms of rarity, where a species classified as NSS was considered most rare*.*

Table 1. List of plant species identified to species level found across the 86 sites with their rarity classification across three different measures of rarity with 1 being rare and 0 being common for abundance amd distribution and NSS for Rabinowitz’s measure of rarity.

| *Species Found* | Rare by Abundance | Rare by Distribution | Rare by Rabinowitz | Serotiny | Fire response | Seed storage location | Life history |  |
| --- | --- | --- | --- | --- | --- | --- | --- | --- |
| *Acacia implexa* | 0 | 0 | NSU | not serotinous | resprouts | soil | perennial |  |
| *Acacia longifolia* | 0 | 0 | NSU | not serotinous | fire killed | soil | perennial |  |
| *Acacia longissima* | 0 | 0 | NSU | not serotinous | fire killed | soil | perennial |  |
| *Acacia suaveolens* | 0 | 0 | NSU | not serotinous | fire killed | soil | perennial |  |
| *Acacia termi lis* | 0 | 0 | NSU | not serotinous | fire killed | soil | perennial |  |
| *Acacia ulicifolia* | 0 | 0 | NSU | not serotinous | fire killed | soil | perennial |  |
| *Acianthus fornicatus* | 1 | 1 | NSS |  | resprouts | soil | perennial |  |
| *Acianthus pusillus* | 0 | 1 | NSS |  | resprouts |  | perennial |  |
| *Acmena smithii* | 1 | 1 | NSS |  | resprouts |  | perennial |  |
| *Actinotus helianthi* | 0 | 0 | NSS |  | fire killed | soil | perennial |  |
| *Actinotus minor* | 0 | 0 | NSU |  | fire killed resprouts | soil | perennial |  |
| *Allocasuari distyla* | 0 | 0 | NSS | serotinous | fire killed | canopy | perennial |  |
| *Allocasuari littoralis* | 0 | 0 | NSS | serotinous | fire killed | canopy | perennial |  |
| *Aotus ericoides* | 0 | 1 | NSS | not serotinous | resprouts | soil | perennial |  |
| *Austrostipa mollis* | 0 | 1 | NSS |  | resprouts |  | perennial |  |
| *Austrostipa pubescens* | 0 | 0 | NSU |  | resprouts | soil | perennial |  |
| *Baeckea brevifolia* | 0 | 1 | NSS |  | fire killed | soil | perennial |  |
| *Baeckea imbricata* | 0 | 0 | NSS |  | resprouts | soil | perennial |  |
| *Baeckea linifolia* | 0 | 1 | NSS |  | resprouts | soil | perennial |  |
| *Baloskion tetraphyllum* | 0 | 0 | NSU |  | resprouts |  | perennial |  |
| *Banksia ericifolia* | 0 | 0 | NSU | serotinous | fire killed |  | perennial |  |
| *Banksia integrifolia* | 0 | 0 | NSU | serotinous | resprouts |  | perennial |  |
| *Banksia paludosa* | 0 | 0 | NSU |  | resprouts | canopy | perennial |  |
| *Banksia serrata* | 0 | 0 | NSU | serotinous | resprouts | canopy | perennial |  |
| *Bauera rubioides* | 0 | 0 | NSU | not serotinous | resprouts | soil | perennial |  |
| *Baumea acuta* | 0 | 0 | NSU |  | resprouts |  | perennial |  |
| *Baumea juncea* | 0 | 0 | NSU |  | resprouts |  | perennial |  |
| *Billardiera scandens* | 0 | 0 | NSU | not serotinous | resprouts | soil | perennial |  |
| *Blandfordia nobilis* | 0 | 1 | NSS |  | resprouts |  | perennial |  |
| *Blechnum ambiguum* | 1 | 1 | NSS |  | resprouts |  | perennial |  |
| *Boronia barkeria* | 0 | 0 | NSS |  | resprouts | soil | perennial |  |
| *Boronia pin ta* | 0 | 0 | NSU | not serotinous | resprouts | soil | perennial |  |
| *Boronia thujo* | 0 | 1 | NSS |  | fire killed | soil | perennial |  |
| *Bossiaea ensata* | 0 | 0 | NSU | not serotinous | resprouts | soil | perennial |  |
| *Bossiaea heterophylla* | 0 | 0 | NSU | not serotinous | resprouts | soil | perennial |  |
| *Bossiaea scolopendria* | 1 | 1 | NSS | not serotinous | resprouts | soil | perennial |  |
| *Brachychiton acerifolius* | 1 | 1 | NSS | not serotinous | resprouts |  | perennial |  |
| *Breynia oblongifolia* | 0 | 0 | NSS | not serotinous | resprouts | soil | perennial |  |
| *Burchardia umbellata* | 1 | 0 | NSU |  | resprouts |  | perennial |  |
| *Caladenia carnea* | 0 | 0 | NSU |  | resprouts |  | perennial |  |
| *Callicoma serratifolia* | 1 | 1 | NSS | not serotinous | resprouts | soil | perennial |  |
| *Callistemon citrinus* | 0 | 0 | NSU |  | resprouts | canopy | perennial |  |
| *Calochlae dubia* | 0 | 0 | NSU |  | resprouts |  | perennial |  |
| *Cassytha glabella* | 0 | 0 | NSU | not serotinous | fire killed | soil | perennial |  |
| *Cassytha pubescens* | 1 | 1 | NSS | not serotinous | fire killed | soil | perennial |  |
| *Casuari glauca* | 0 | 0 | NSU |  | resprouts | canopy | perennial |  |
| *Caustis flexuosa* | 0 | 0 | NSU |  | fire killed resprouts | soil | perennial |  |
| *Caustis pentandra* | 0 | 0 | NSU |  | resprouts | soil | perennial |  |
| *Caustis recurvata* | 0 | 0 | NSU |  | resprouts |  | perennial |  |
| *Cenchrus clandestinus* | 0 | 1 | NSS |  |  |  | perennial |  |
| *Ceratopetalum gummiferum* | 0 | 0 | NSS | not serotinous | resprouts |  | perennial |  |
| *Chordifex fastigiatus* | 0 | 0 | NSS |  | resprouts |  | perennial |  |
| *Chorizema parviflorum* | 1 | 1 | NSS |  | resprouts | soil | perennial |  |
| *Chrysanthemoides monilifera* | 0 | 0 | NSS |  | fire killed |  | perennial |  |
| *Cissus hypoglauca* | 0 | 0 | NSS | not serotinous | resprouts |  | perennial |  |
| *Clematis aristata* | 0 | 0 | NSS | not serotinous | resprouts |  | perennial |  |
| *Commeli cyanea* | 0 | 0 | NSS |  | fire killed resprouts |  | perennial |  |
| *Commersonia hermanniifolia* | 0 | 1 | NSS |  |  |  | perennial |  |
| *Conyza sumatrensis* | 1 | 1 | NSS |  | fire killed | soil | annual |  |
| *Coronidium elatum* | 0 | 0 | NSU |  | fire killed |  |  |  |
| *Correa reflexa var. reflexa* | 0 | 1 | NSS |  | resprouts | soil | perennial |  |
| *Corymbia gummifera* | 0 | 0 | NSU |  | resprouts | canopy | perennial |  |
| *Cryptandra ericoides* | 1 | 1 | NSS | not serotinous | fire killed | soil | perennial |  |
| *Cryptostylis erecta* | 1 | 0 | NSU |  | resprouts |  | perennial |  |
| *Cyathochaeta diandra* | 0 | 0 | NSU |  | resprouts | soil | perennial |  |
| *Cyperus gracilis* | 0 | 0 | NSS |  | resprouts |  | perennial |  |
| *Dampiera purpurea* | 1 | 0 | NSU |  | resprouts |  | perennial |  |
| *Dampiera stricta* | 0 | 0 | NSU |  | resprouts | soil | perennial |  |
| *Darwinia camptostylis* | 0 | 1 | NSS |  | resprouts |  | perennial |  |
| *Darwinia leptantha* | 0 | 0 | NSU |  | fire killed | soil | perennial |  |
| *Desdemonium gunni* | 0 | 1 | NSS |  | resprouts | soil | perennial |  |
| *Desmodium brachypodum* | 0 | 0 | NSU | not serotinous | resprouts | soil | perennial |  |
| *Desmodium rhytidophyllum* | 0 | 0 | NSU | not serotinous | resprouts | soil | perennial |  |
| *Desmodium varians* | 0 | 0 | NSS | not serotinous | resprouts | soil | perennial |  |
| *Dianella caerulea* | 0 | 0 | NSU |  | resprouts |  | perennial |  |
| *Dichondra repens* | 0 | 0 | NSU |  | resprouts |  | perennial |  |
| *Dillwynia elegans* | 0 | 1 | NSS |  | fire killed | soil | perennial |  |
| *Dillwynia floribunda* | 0 | 0 | NSU | not serotinous | fire killed | soil | perennial |  |
| *Dillwynia floribunda var. floribunda* | 0 | 0 | NSU | not serotinous | fire killed | soil | perennial |  |
| *Dillwynia glaberrima* | 0 | 0 | NSU |  | resprouts | soil | perennial |  |
| *Dillwynia retorta* | 0 | 0 | NSU | not serotinous | fire killed |  | perennial |  |
| *Dodo ea triquetra* | 0 | 1 | NSS | not serotinous | resprouts | soil | perennial |  |
| *Drosera bi ta* | 0 | 0 | NSS | not serotinous | resprouts |  | perennial |  |
| *Drosera peltata* | 0 | 0 | NSU | not serotinous | resprouts |  | perennial |  |
| *Echinopogon ovatus* | 1 | 1 | NSS |  | resprouts | soil | perennial |  |
| *Ehrharta erecta* | 0 | 1 | NSS |  | resprouts |  | annual |  |
| *Elaeocarpus reticulatus* | 0 | 0 | NSU | not serotinous | resprouts |  | perennial |  |
| *Elaeodendron australe* | 1 | 1 | NSS |  | resprouts |  | perennial |  |
| *Empodisma minus* | 0 | 0 | NSU |  | resprouts |  | perennial |  |
| *Entolasia margi ta* | 0 | 0 | WSU |  | resprouts | soil | perennial |  |
| *Entolasia stricta* | 0 | 0 | NSU |  | resprouts | soil | perennial |  |
| *Epacris longiflora* | 0 | 1 | NSS |  | fire killed | soil | perennial |  |
| *Epacris microphylla* | 0 | 0 | NSU |  | resprouts | soil | perennial |  |
| *Epacris microphylla var. microphylla* | 0 | 0 | NSU |  | fire killed |  |  |  |
| *Epacris obtusifolia* | 0 | 0 | NSS |  | fire killed | soil | perennial |  |
| *Epacris pulchella* | 0 | 0 | NSU |  | fire killed | soil | perennial |  |
| *Eragrostis brownii* | 1 | 1 | NSS |  | resprouts |  | perennial |  |
| *Eucalyptus botryoides* | 1 | 0 | NSU |  | resprouts | canopy | perennial |  |
| *Eucalyptus burgessia* | 0 | 1 | NSS |  | resprouts | canopy | perennial |  |
| *Eucalyptus pilularis* | 0 | 0 | NSU |  | resprouts | canopy | perennial |  |
| *Eucalyptus sclerophylla* | 1 | 0 | NSS |  | resprouts | canopy | perennial |  |
| *Eurychorda compla ta* | 0 | 0 | NSS |  | fire killed resprouts | soil | perennial |  |
| *Eustrephus latifolius* | 0 | 0 | NSS |  | resprouts |  | perennial |  |
| *Ficinia nodosa* | 0 | 0 | NSS |  | resprouts | soil | perennial |  |
| *Gahnia clarkei* | 0 | 0 | NSU |  | resprouts |  | perennial |  |
| *Gahnia sieberia* | 0 | 0 | NSS |  | resprouts | soil | perennial |  |
| *Galium gaudichaudii* | 0 | 0 | NSS |  | fire killed |  | perennial |  |
| *Galium propinquum* | 0 | 0 | NSU |  | resprouts |  | perennial |  |
| *Gastrodia sesamoides* | 0 | 1 | NSS |  | resprouts |  | perennial |  |
| *Geranium homeanum* | 0 | 0 | NSU |  | fire killed resprouts |  | perennial |  |
| *Gleichenia dicarpa* | 0 | 0 | NLU |  | resprouts |  | perennial |  |
| *Glycine clandesti* | 0 | 0 | NSS | not serotinous | fire killed | soil | perennial |  |
| *Glycine tabaci* | 0 | 0 | NSS |  | resprouts | soil | perennial |  |
| *Gompholobium grandiflorum* | 0 | 1 | NSS | not serotinous | resprouts | soil | perennial |  |
| *Gompholobium latifolium* | 0 | 0 | NSS | not serotinous | fire killed | soil | perennial |  |
| *Gonocarpus micranthus* | 0 | 0 | NSS |  | resprouts | soil | perennial |  |
| *Gonocarpus teucrioides* | 0 | 0 | NSU |  | fire killed | soil | perennial |  |
| *Goodenia bellidifolia* | 0 | 1 | NSS |  | resprouts |  | perennial |  |
| *Goodenia hederacea* | 1 | 1 | NSS |  | resprouts |  | perennial |  |
| *Goodenia stelligera* | 1 | 1 | NSS |  | fire killed resprouts | soil | perennial |  |
| *Grevillea macleaya* | 0 | 0 | NSS |  | fire killed |  | perennial |  |
| *Haemodorum corymbosum* | 0 | 0 | NSU |  | resprouts |  | perennial |  |
| *Haemodorum planifolium* | 0 | 0 | NSU |  | resprouts |  | perennial |  |
| *Hakea dactyloides* | 0 | 0 | NSU | serotinous | resprouts | canopy | perennial |  |
| *Hakea sericea* | 0 | 0 | NSS | serotinous | fire killed | canopy | perennial |  |
| *Hakea teretifolia* | 0 | 0 | NSU | serotinous | fire killed | canopy | perennial |  |
| *Hardenbergia violacea* | 0 | 0 | NSU | not serotinous | resprouts | soil | perennial |  |
| *Hibbertia dentata* | 0 | 0 | NSS |  | resprouts | soil | perennial |  |
| *Hibbertia diffusa* | 0 | 0 | NSU |  | fire killed | soil | perennial |  |
| *Hibbertia empetrifolia* | 0 | 0 | NSU |  | resprouts |  | perennial |  |
| *Hibbertia fasciculata* | 0 | 1 | NSS |  | fire killed | soil | perennial |  |
| *Hibbertia hermanniifolia* | 0 | 1 | NSS |  | fire killed | soil | perennial |  |
| *Hibbertia linearis* | 0 | 0 | NSU |  | fire killed | soil | perennial |  |
| *Hibbertia obtusifolia* | 1 | 1 | NSS |  | resprouts | soil | perennial |  |
| *Hibbertia riparia* | 0 | 0 | NSU |  | resprouts | soil | perennial |  |
| *Hibbertia scandens* | 0 | 0 | NSU |  | resprouts | soil | perennial |  |
| *Hibbertia virgata* | 1 | 0 | NSU |  | resprouts |  | perennial |  |
| *Histiopteris incisa* | 0 | 1 | NSS |  | fire killed resprouts |  | perennial |  |
| *Homalanthus populifolius* | 1 | 1 | NSS |  | fire killed resprouts |  | perennial |  |
| *Hovea linearis* | 0 | 0 | NSS | not serotinous | resprouts | soil | perennial |  |
| *Hybanthus monopetalus* | 0 | 0 | NSU |  | fire killed resprouts | soil | perennial |  |
| *Hybanthus vernonii* | 1 | 1 | NSS |  | fire killed resprouts |  | perennial |  |
| *Hydrocotyle bo riensis* | 0 | 0 | NSS |  | resprouts |  | perennial |  |
| *Hydrocotyle peduncularis* | 0 | 0 | NSS |  | resprouts |  | perennial |  |
| *Hypochaeris radicata* | 0 | 1 | NSU |  | fire killed resprouts |  | annual |  |
| *Hypolae fastigiata* | 0 | 0 | NSU |  | resprouts | soil | perennial |  |
| *Hypolepis muelleri* | 0 | 1 | NSU |  | resprouts |  | perennial |  |
| *Hypoxis hygrometrica var. hygrometrica* | 1 | 1 | NSS |  | resprouts |  | perennial |  |
| *Imperata cylindrica var. major* | 0 | 0 | WSU |  | resprouts |  |  |  |
| *Isopogon anemonifolius* | 0 | 0 | NSU | serotinous | resprouts | canopy | perennial |  |
| *Isopogon anethifolius* | 1 | 0 | NSU | serotinous | resprouts | canopy | perennial |  |
| *Juncus pallidus* | 0 | 1 | NLS |  | resprouts | soil | perennial |  |
| *Juncus usitatus* | 0 | 0 | NLS |  | resprouts |  | perennial |  |
| *Kennedia rubicunda* | 0 | 0 | NSU | not serotinous | resprouts | soil | perennial |  |
| *Kunzea capitata* | 0 | 0 | NSS |  | fire killed | soil | perennial |  |
| *Lagenifera stipitata* | 0 | 0 | NSS |  | resprouts |  | perennial |  |
| *Lagenophora stipitata* | 1 | 1 | NSS |  | resprouts |  | perennial |  |
| *Lambertia formosa* | 0 | 0 | NSU | serotinous | resprouts | canopy | perennial |  |
| *Leionema diosmeum* | 0 | 1 | NLS |  | fire killed | soil | perennial |  |
| *Leptocarpus te x* | 0 | 0 | NSU |  | resprouts | soil | perennial |  |
| *Leptomeria acida* | 0 | 0 | NSU | not serotinous | fire killed | soil | perennial |  |
| *Lepyrodia scariosa* | 0 | 0 | NSU |  | resprouts | soil | perennial |  |
| *Leucopogon ericoides* | 0 | 0 | NSU |  | fire killed | soil | perennial |  |
| *Leucopogon esquamatus* | 0 | 0 | NSS |  | fire killed | soil | perennial |  |
| *Leucopogon lanceolatus* | 0 | 0 | NSU |  | resprouts |  | perennial |  |
| *Leucopogon parviflorus* | 1 | 1 | NSS |  | resprouts | soil | perennial |  |
| *Lindsaea linearis* | 0 | 0 | NSU |  | resprouts |  | perennial |  |
| *Livisto australis* | 0 | 1 | NSS |  | resprouts |  | perennial |  |
| *Lobelia anceps* | 0 | 1 | NSS |  | fire killed resprouts |  | perennial |  |
| *Lobelia dentata* | 1 | 0 | NSU |  | resprouts |  | annual |  |
| *Lobelia purpurascens* | 0 | 0 | NSS |  | resprouts |  | perennial |  |
| *Lomandra cylindrica* | 0 | 0 | NSU |  | resprouts | soil |  |  |
| *Lomandra filiformis* | 0 | 1 | NSS |  | resprouts | soil | perennial |  |
| *Lomandra glauca* | 0 | 0 | NSU |  | resprouts | soil | perennial |  |
| *Lomandra gracilis* | 0 | 0 | NSU |  | resprouts | soil | perennial |  |
| *Lomandra longifolia* | 0 | 0 | WSU |  | resprouts | soil | perennial |  |
| *Lomandra obliqua* | 0 | 0 | NSU |  | fire killed resprouts | soil | perennial |  |
| *Lomatia ilicifolia* | 0 | 0 | NSS |  | resprouts | soil | perennial |  |
| *Lycopodium deuterodensum* | 0 | 1 | NLS |  | resprouts |  | perennial |  |
| *Lycopodium laterale* | 0 | 1 | NSS |  | resprouts |  |  |  |
| *Lysimachia arvensis* | 1 | 1 | NSS |  |  |  | perennial |  |
| *Marsdenia rostrata* | 0 | 0 | NSU |  | resprouts |  | perennial |  |
| *Marsdenia suaveolens* | 0 | 0 | NSS |  | resprouts |  | perennial |  |
| *Melaleuca capitata* | 0 | 1 | NLS |  | resprouts |  | perennial |  |
| *Melaleuca li riifolia* | 0 | 1 | NSS |  | resprouts | canopy soil | perennial |  |
| *Melaleuca sieberi* | 0 | 1 | NSS |  | resprouts | canopy soil | perennial |  |
| *Melaleuca squarrosa* | 0 | 0 | NSS |  | resprouts | canopy | perennial |  |
| *Melaleuca thymifolia* | 0 | 0 | NSS |  | resprouts | canopy | perennial |  |
| *Melaleuca thymifolia* | 1 | 1 | NSS |  |  |  |  |  |
| *Microlae stipoides var. stipoides* | 0 | 0 | NSU |  | resprouts | soil | perennial |  |
| *Mirbelia rubiifolia* | 0 | 0 | NSS | not serotinous | fire killed resprouts | soil | perennial |  |
| *Mitrasacme polymorpha* | 1 | 0 | NSU |  | fire killed resprouts | soil | perennial |  |
| *Monotoca elliptica* | 0 | 0 | NSU |  | fire killed |  | perennial |  |
| *Monotoca scoparia* | 0 | 0 | NSU | not serotinous | resprouts | soil | perennial |  |
| *Myoporum acumi tum* | 0 | 0 | NSU |  | resprouts |  | perennial |  |
| *Notelaea longifolia* | 0 | 0 | NSS |  | resprouts |  | perennial |  |
| *Notelaea longifolia forma longifolia* | 0 | 0 | NSS |  | resprouts |  |  |  |
| *Notelaea ovata* | 0 | 0 | NSS |  | resprouts |  | perennial |  |
| *Notelaea venosa* | 0 | 1 | NSS |  | resprouts |  | perennial |  |
| *Opercularia diphylla* | 0 | 0 | NSU |  | fire killed resprouts | soil | perennial |  |
| *Opercularia varia* | 0 | 1 | NSS |  | fire killed | soil | perennial |  |
| *Oplismenus aemulus* | 0 | 0 | NSS |  | resprouts |  | perennial |  |
| *Oplismenus imbecillis* | 1 | 1 | NSS |  | resprouts |  | perennial |  |
| *Oxalis articulata* | 0 | 1 | NSS |  |  |  | perennial |  |
| *Oxalis corniculata* | 0 | 1 | NSS |  | resprouts | soil | perennial |  |
| *Oxalis peren ns* | 0 | 0 | NSS |  | resprouts |  | annual |  |
| *Pandorea pandora* | 0 | 1 | NSS |  | resprouts |  | perennial |  |
| *Parsonsia straminea* | 0 | 0 | NSU |  | resprouts |  | perennial |  |
| *Patersonia fragilis* | 0 | 1 | NSS |  | resprouts | soil | perennial |  |
| *Patersonia glabrata* | 0 | 1 | NSU |  | resprouts | soil | perennial |  |
| *Patersonia sericea* | 0 | 0 | NSU |  | resprouts | soil | perennial |  |
| *Pelargonium inodorum* | 1 | 1 | NSS | not serotinous | fire killed |  | annual |  |
| *Persoonia levis* | 0 | 0 | NSU | not serotinous | resprouts | soil | perennial |  |
| *Persoonia linearis* | 0 | 0 | NSU | not serotinous | resprouts | soil | perennial |  |
| *Petrophile pedunculata* | 1 | 1 | NSS |  | resprouts | canopy | perennial |  |
| *Petrophile pulchella* | 0 | 0 | NSU | serotinous | fire killed | canopy | perennial |  |
| *Petrophile sessilis* | 0 | 0 | NSU |  | fire killed | canopy | perennial |  |
| *Philotheca buxifolia* | 0 | 0 | NSU |  | fire killed | soil | perennial |  |
| *Phyllota phylicoides* | 0 | 1 | NSS | not serotinous | fire killed resprouts | soil | perennial |  |
| *Pimelea ligustri* | 0 | 1 | NSU |  | fire killed |  | perennial |  |
| *Pimelea linifolia* | 0 | 0 | NSU |  | resprouts | soil | perennial |  |
| *Platylobium formosum* | 0 | 0 | NSU | not serotinous | fire killed | soil | perennial |  |
| *Platysace lanceolata* | 1 | 1 | NSU |  | resprouts | soil | perennial |  |
| *Poa labillardieri* | 0 | 0 | NSU |  | resprouts |  | perennial |  |
| *Polyscias sambucifolia* | 1 | 1 | NSS |  | resprouts | soil | perennial |  |
| *Pomax umbellata* | 0 | 1 | NSS |  | fire killed | soil | perennial |  |
| *Poranthera ericifolia* | 1 | 0 | NSU | not serotinous | fire killed | soil | perennial |  |
| *Poranthera microphylla* | 0 | 0 | NSU | not serotinous | fire killed | soil | annual |  |
| *Prasophyllum brevilabre* | 1 | 1 | NSS |  | resprouts |  | perennial |  |
| *Psychotria loniceroides* | 1 | 1 | NSS |  | resprouts |  | perennial |  |
| *Pteridium esculentum* | 0 | 0 | WSU |  | resprouts | soil | perennial |  |
| *Ptilothrix deusta* | 0 | 0 | NSS |  | resprouts | soil | perennial |  |
| *Pultenea daphnoides* | 0 | 0 | NSU | not serotinous | fire killed | soil | perennial |  |
| *Pultenea rosmarinifolia* | 0 | 0 | NSU | not serotinous | fire killed | soil | perennial |  |
| *Pultenea villifera* | 0 | 0 | NSS |  | fire killed | soil | perennial |  |
| *Pultenea villosa* | 0 | 1 | NSS |  | fire killed | soil | Perennial |  |
| *Sarcopetalum harveyanum* | 0 | 1 | NSS | Not serotinous | resprouts |  | perennial | |
| *Scaevola calendulacea* | 1 | 1 | NSS |  | Fire killed |  | perennial | |
| *Scaevola ramosissima* | 0 | 0 | NSU |  | resprouts | soil | perennial | |
| *Schelhammera undulata* | 0 | 0 | NSS |  | resprouts |  | perennial | |
| *Schizaea bifida* | 0 | 0 | NSU |  | resprouts |  | perennial | |
| *Schizaea dichotoma* | 1 | 1 | NSS |  | resprouts |  | perennial | |
| *Schizaea fistulosa* | 0 | 1 | NSS |  | resprouts |  | perennial | |
| *Schoenus ericetorum* | 0 | 1 | NSS |  | resprouts | soil | perennial | |
| *Schoenus lepidosperma subsp. pachylepis* | 0 | 1 | NLS |  | resprouts |  |  | |
| *Selaginella uliginosa* | 0 | 0 | NSU |  | resprouts |  | perennial | |
| *Smilax glyciphylla* | 0 | 0 | NSU |  | resprouts |  | perennial | |
| *Solanum nigrum* | 1 | 1 | NSS |  | fire killed | none | biennial | |
| *Solanum prinophyllum* | 0 | 0 | NSU |  | fire killed | soil | perennial | |
| *Solanum stelligerum* | 1 | 1 | NSS |  | fire killed | soil | perennial | |
| *Sowerbaea juncea* | 0 | 1 | NSS |  | resprouts |  | perennial | |
| *Sphaerolobium vimineum* | 0 | 0 | NSS | Not serotinous | fire killed | soil | perennial | |
| *Sprengelia incarta* | 0 | 0 | NSS |  | fire killed | soil | perennial | |
| *Stackhousia nuda* | 0 | 1 | NSS | Not serotinous | fire killed resprouts | soil | perennial | |
| *Stephania japonica var. discolor* | 0 | 0 | NSS |  | resprouts |  | perennial | |
| *Stylidium laricifolium* | 0 | 1 | NSU |  | fire killed |  | perennial | |
| *Stylidium lineare* | 0 | 1 | NSS |  | resprouts | soil | perennial | |
| *Styphelia triflora* | 0 | 0 | NSU |  | fire killed | soil | perennial | |
| *Styphelia tubiflora* | 0 | 1 | NSS |  | fire killed | soil | perennial | |
| *Syncarpia glomulifera* | 0 | 1 | NSU |  | Not fire killed does not resprout |  | perennial | |
| *Synoum glandulosum* | 0 | 0 | NSU | Not serotinous | resprouts |  | perennial | |
| *Syzygium paniculatum* | 0 | 1 | NSS |  | fire killed, resprouts | perennial |  |  |
| *Telopea speciosissima* | 0 | 0 | NSU | Not serotinous | resprouts |  | perennial | |
| *Tetratheca thymifolia* | 1 | 1 | NSS | not serotinous | fire killed | soil | perennial | |
| *Thelionema umbellatum* | 1 | 1 | NSS |  | resprouts |  | perennial | |
| *Themeda australis* | 0 | 0 | NSU |  | resprouts |  | perennial | |
| *Themeda triandra* | 0 | 1 | NSS |  | resprouts | soil | perennial | |
| *Thysanotus juncifolius* | 0 | 0 | NSU |  | resprouts |  | annual | |
| *Thysanotus tuberosus* | 0 | 0 | NSU |  | resprouts |  | perennial | |
| *Tristaniopsis colli* | 1 | 1 | NSS |  | resprouts | canopy | perennial | |
| *Urtica incisa* | 0 | 1 | NLS |  | fire killed | soil | perennial | |
| *Utricularia dichotoma* | 0 | 1 | NSS |  | resprouts |  | perennial | |
| *Utricularia lateriflora* | 1 | 1 | NSS |  | resprouts |  | annual | |
| *Utricularia uliginosa* | 1 | 1 | NSS |  |  |  | perennial | |
| *Utricularia uniflora* | 1 | 1 | NSS |  | resprouts |  | perennial | |
| *Veronica calyci* | 0 | 1 | NSU |  | fire killed |  | perennial | |
| *Vimiria juncea* | 0 | 0 | NSU | not serotinous | fire killed | soil | perennial | |
| *Viola hederacea* | 0 | 0 | NSU |  | resprouts | soil | perennial | |
| *Woollsia pungens* | 0 | 0 | NSS |  | fire killed | soil | perennial | |
| *Xanthorrhoea australis* | 0 | 1 | NSS |  | resprouts |  | perennial | |
| *Xanthorrhoea minor subsp. lutea* | 0 | 1 | NSS |  | resprouts |  | perennial | |
| *Xanthorrhoea resinifera* | 0 | 0 | NSU |  | resprouts |  | perennial | |
| *Xanthorrhoea resinosa* | 0 | 1 | NSS |  | resprouts |  | perennial | |
| *Xanthosia pilosa* | 0 | 0 | NSU |  | resprouts | soil | perennial | |
| *Xanthosia tridentata* | 0 | 0 | NSS |  | fire killed | soil | perennial | |
| *Xyris gracilis* | 1 | 1 | NSS |  | resprouts | soil | perennial | |
| *Xyris operculata* | 0 | 0 | NSS |  | resprouts | soil | perennial | |
| *Zieria pilosa* | 0 | 1 | NSS |  | fire killed | soil | perennial | |
| *Zieria smithii* | 0 | 0 | NSS |  | resprouts | soil | perennial | |
| *Zoysia macrantha* | 0 | 0 | NSS |  | resprouts |  | perennial | |

**Table 2.** Rare species richness for each vegetation community across the three rarity categorisations used.

| Richness measure |  | Vegetation community | | |  |
| --- | --- | --- | --- | --- | --- |
| Rare species richness |  | Woodland | Heath | Forest | Overall (across all three communities) |
| Abundance | | 55 | 54 | 48 | 133 |
| Distribution | | 83 | 85 | 74 | 221 |
| Rabinowitz | NLS | 1 | 7 | 2 | 10 |
|  | NLU | 1 | 1 | 0 | 1 |
|  | NSU | 163 | 140 | 98 | 179 |
|  | WSU (most common) | 4 | 4 | 4 | 4 |
|  | NSS (rare) | 81 | 91 | 89 | 261 |
| Total species richness |  | 250 | 243 | 193 | 455 |

# Electronic Supplemental Material 4

Table 1. Negative binomial generalized linear model outputs testing for the association between fire regime variables across the 86 sites surveyed with species richness and rare species richness. Raw estimates, standardised regression coefficients and estimated 95% confidence intervals are shown. The reference level for vegetation community (VegType) and vegetation x fire interactions (against which other types are compared) is forest. Values in bold indicate significance, with the cut-off for statistical significance being P < 0.05.

|  | **Total species richness** | | | **Abundance** | | | **Distribution** | | | | **Rabinowitz** | | |
| --- | --- | --- | --- | --- | --- | --- | --- | --- | --- | --- | --- | --- | --- |
| *Coefficient* | *Log-Mean* | *Conf. Int (95%)* | *P-Value* | *Log-Mean* | *Conf. Int (95%)* | *P-Value* | *Log-Mean* | *Conf. Int (95%)* | *P-Value* | *Log-Mean* | | *Conf. Int (95%)* | *P-Value* |
| Intercept | 3.51 | 3.17 – 3.85 | **<0.001** | 0.53 | 0.20 – 0.86 | **0.002** | 0.88 | 0.41 – 1.35 | **<0.001** | 2.46 | | 2.22 – 2.71 | **<0.001** |
| VegType [heath] | 0.49 | 0.00 – 0.98 | **0.048** | 0.33 | -0.10 – 0.76 | 0.128 | 0.76 | 0.17 – 1.35 | **0.011** | -0.22 | | -0.80 – 0.37 | 0.465 |
| VegType [woodland] | -0.58 | -1.02 – -0.14 | **0.010** | 0.80 | 0.37 – 1.23 | **<0.001** | 0.70 | 0.07 – 1.34 | **0.031** | -1.09 | | -1.64 – -0.53 | **<0.001** |
| Fire Frequency | -0.03 | -0.12 – 0.05 | 0.453 |  |  |  |  |  |  | -0.12 | | -0.22 – -0.02 | **0.017** |
| Short Interval | 0.18 | 0.00 – 0.37 | **0.045** |  |  |  |  |  |  | 0.40 | | 0.10 – 0.71 | **0.010** |
| Log (Time since fire) | -0.06 | -0.15 – 0.02 | 0.132 |  |  |  | -0.06 | -0.26 – 0.14 | 0.565 |  | |  |  |
| VegType [heath]*Fire Frequency | -0.11 | -0.24 – 0.01 | 0.076 |  |  |  |  |  |  | 0.11 | | -0.06 – 0.29 | 0.211 |
| VegType [woodland]*Fire Frequency | 0.27 | 0.16 – 0.37 | **<0.001** |  |  |  |  |  |  | 0.31 | | 0.12 – 0.49 | **0.001** |
| VegType [heath]*Short Interval | -0.01 | -0.32 – 0.31 | 0.964 |  |  |  |  |  |  | -0.06 | | -0.62 – 0.51 | 0.846 |
| VegType [woodland]*Short Interval | -0.68 | -0.96 – -0.40 | **<0.001** |  |  |  |  |  |  | -1.18 | | -1.84 – -0.51 | **0.001** |
| VegType [heath]*log (Time since fire) | -0.01 | -0.12 – 0.09 | 0.790 |  |  |  | -0.23 | -0.54 – 0.07 | 0.129 |  | |  |  |
| VegType [woodland]*log(Time since fire) | 0.17 | 0.07 – 0.27 | **0.001** |  |  |  | -0.02 | -0.30 – 0.25 | 0.880 |  | |  |  |
| Elevation |  |  |  | 0.00 | -0.00 – 0.01 | 0.264 |  |  |  |  | |  |  |
| Aspect-Eastings |  |  |  | 0.21 | -0.05 – 0.47 | 0.116 |  |  |  |  | |  |  |

Table 2. Estimated marginal means of trends for models examining the species richness and rare species richness with fire regime variables, calculated using the R package emmeans ^32^.

| Richness measure | Vegetation community | Fire frequency trend | Log (Time since fire) trend | Short Interval trend | Asymptotic Lower confidence interval | Asymptotic Upper confidence interval |
| --- | --- | --- | --- | --- | --- | --- |
| Species richness | forest | -0.033 |  |  | -0.119 | 0.053 |
|  | heath | -0.145 |  |  | -0.233 | -0.056 |
|  | woodland | 0.232 |  |  | 0.165 | 0.299 |
|  | forest |  | -0.063 |  | -0.146 | 0.019 |
|  | heath |  | -0.078 |  | -0.142 | -0.013 |
|  | woodland |  | 0.109 |  | 0.052 | 0.165 |
|  | forest |  |  | 0.185 | 0.004 | 0.366 |
|  | heath |  |  | 0.178 | -0.082 | 0.437 |
|  | woodland |  |  | -0.493 | -0.707 | -0.279 |
| Distribution | forest |  | -0.059 |  | -0.258 | 0.141 |
|  | heath |  | -0.293 |  | -0.52 | -0.065 |
|  | woodland |  | -0.08 |  | -0.27 | 0.11 |
| Rabinowitz | forest |  |  | 0.401 | 0.096 | 0.706 |
|  | heath |  |  | 0.345 | -0.127 | 0.818 |
|  | woodland |  |  | -0.774 | -1.366 | -0.182 |
|  | forest | -0.12 |  |  | -0.22 | -0.021 |
|  | heath | -0.006 |  |  | -0.156 | 0.144 |
|  | woodland | 0.185 |  |  | 0.028 | 0.342 |

# Electronic Supplemental Material 5

Table 1. The model outputs testing for the association between fire regime variables across the 86 sites surveyed with the proportion of rare species in an assemblage (Table 1). Raw estimates, standardised regression coefficients and estimated 95% confidence intervals are shown. The reference level for vegetation community (VegType) and vegetation x fire interactions (against which other types are compared) is forest. Values in bold indicate significance, with the cut-off for statistical significance being P < 0.05.

|  | **Abundance** | | | **Distribution** | | | **Rabinowitz** | | |
| --- | --- | --- | --- | --- | --- | --- | --- | --- | --- |
| *Log-odds* | *Log-Odds* | *Conf. Int (95%)* | *P-Value* | *Log-Odds* | *Conf. Int (95%)* | *P-Value* | *Log-Odds* | *Conf. Int (95%)* | *P-Value* |
| Intercept | -2.66 | -2.89 – -2.42 | **<0.001** | -2.52 | -2.92 – -2.12 | **<0.001** | -0.16 | -0.42 – 0.10 | 0.220 |
| VegType [heath] | 0.39 | 0.05 – 0.73 | **0.025** | 0.77 | 0.29 – 1.25 | **0.002** | -0.57 | -1.18 – 0.05 | 0.071 |
| VegType [woodland] | 0.41 | 0.08 – 0.73 | **0.013** | 0.41 | -0.10 – 0.92 | 0.114 | -1.08 | -1.62 – -0.54 | **<0.001** |
| log (Time since fire) |  |  |  | -0.06 | -0.24 – 0.12 | 0.517 |  |  |  |
| Aspect-Northings |  |  |  | -0.20 | -0.48 – 0.08 | 0.169 |  |  |  |
| VegType [heath]*log (Time since fire) |  |  |  | -0.23 | -0.48 – 0.03 | 0.083 |  |  |  |
| VegType [woodland]* log (Time since fire) |  |  |  | -0.05 | -0.28 – 0.17 | 0.650 |  |  |  |
| Fire Frequency |  |  |  |  |  |  | -0.14 | -0.24 – -0.05 | **0.004** |
| Short Interval |  |  |  |  |  |  | 0.25 | 0.00 – 0.49 | **0.045** |
| Elevation |  |  |  |  |  |  | -0.01 | -0.01 – -0.00 | **<0.001** |
| Aspect-Eastings |  |  |  |  |  |  | -0.13 | -0.27 – 0.01 | 0.062 |
| VegType [heath]*Fire Frequency |  |  |  |  |  |  | 0.24 | 0.07 – 0.41 | **0.006** |
| VegType [woodland]*Fire Frequency |  |  |  |  |  |  | 0.05 | -0.11 – 0.20 | 0.557 |
|  |  | | |  | | |  | | |
|  |  | | |  | | |  | | |

Table 2. Estimated marginal means of trends for models examining the proportion of rare species with relevant fire regime variables , calculated using the R package emmeans ^32^.

| Rarity type | Vegetation community | Log  (Time since fire) trend | Fire frequency trend | Short Interval trend | Asymptotic Lower confidence interval | Asymptotic Upper confidence interval |
| --- | --- | --- | --- | --- | --- | --- |
| Distribution | forest | -0.059 |  |  | -0.236 | 0.119 |
|  | heath | -0.286 |  |  | -0.47 | -0.101 |
|  | woodland | -0.111 |  |  | -0.257 | 0.035 |
| Rabinowitz | forest |  | -0.142 |  | -0.239 | -0.045 |
|  | heath |  | 0.094 |  | -0.052 | 0.24 |
|  | woodland |  | -0.096 |  | -0.228 | 0.037 |
|  | forest |  |  | 0.245 | 0.003 | 0.487 |
|  | heath |  |  | 0.245 | 0.003 | 0.487 |
|  | woodland |  |  | 0.245 | 0.003 | 0.487 |

# Electronic Supplemental Material 6

**Table 1.** Fisher’s exact test results examining if rare species were associated with particular fire response traits, regeneration strategies, and life history compared species not classified as rare.

| Rarity measure | Trait | Fisher’s exact test P-value |
| --- | --- | --- |
| Abundance | Fire response | 0.672 |
|  | Serotiny | 1.000 |
|  | Seed storage location | 0.125 |
|  | Life history | **0.018** |
| Distribution | Fire response | 0.820 |
|  | Serotiny | 0.059 |
|  | Seed storage location | **0.003** |
|  | Life history | 0.149 |
| Rabinowitz | Fire response | 0.728 |
|  | Serotiny | 0.536 |
|  | Seed storage location | 0.340 |
|  | Life history | 1.000 |

**Table 2**. Number and proportion of rare and not rare species within each trait category in the woodland vegetation community.

| **Rarity Measure** | **Number and proportion of species present** | **Rarity** | **Fire response trait** | | | **Serotiny** | | **Seed storage location** | | **Life history** | |
| --- | --- | --- | --- | --- | --- | --- | --- | --- | --- | --- | --- |
|  |  |  | **fire killed** | **fire killed resprouts** | **resprouts** | **not serotinous** | **serotinous** | **canopy** | **soil** | **annual** | **perennial** |
| Abundance | Number of species present | Not Rare | 56 | 7 | 106 | 37 | 11 | 19 | 93 | 2 | 156 |
|  |  | Rare | 3 | 2 | 17 | 3 | 1 | 4 | 6 | 1 | 18 |
|  | Proportion of species with trait present | Not Rare | 0.949 | 0.778 | 0.862 | 0.925 | 0.917 | 0.826 | 0.939 | 0.667 | 0.897 |
|  |  | Rare | 0.051 | 0.222 | 0.138 | 0.075 | 0.083 | 0.174 | 0.061 | 0.333 | 0.103 |
| Distribution | Number of species present | Not Rare | 42 | 7 | 101 | 34 | 12 | 21 | 74 | 3 | 137 |
|  |  | Rare | 17 | 2 | 22 | 6 | 0 | 2 | 25 | 0 | 37 |
|  | Proportion of species with trait present | Not Rare | 0.712 | 0.778 | 0.821 | 0.85 | 1 | 0.913 | 0.747 | 1 | 0.787 |
|  |  | Rare | 0.288 | 0.222 | 0.179 | 0.15 | 0 | 0.087 | 0.253 | 0 | 0.213 |
| Rabinowitz | Number of species present | Not Rare | 37 | 7 | 98 | 31 | 9 | 17 | 69 | 3 | 128 |
|  |  | Rare (NSS) | 22 | 2 | 25 | 9 | 3 | 6 | 30 | 0 | 46 |
|  | Proportion of species with trait present | Not Rare | 0.627 | 0.778 | 0.797 | 0.775 | 0.75 | 0.739 | 0.697 | 1 | 0.736 |
|  |  | Rare (NSS) | 0.373 | 0.222 | 0.203 | 0.225 | 0.25 | 0.261 | 0.303 | 0 | 0.264 |

**Table 3**. Number and proportion of rare and not rare species within each trait category in the forest vegetation community.

| Rarity Measure | Number and proportion of species present | Rarity | Fire response trait | | | | Serotiny | | Seed storage location | | |  | Life history | | |
| --- | --- | --- | --- | --- | --- | --- | --- | --- | --- | --- | --- | --- | --- | --- | --- |
|  |  |  | **fire killed** | **fire killed resprouts** | **not fire killed, does not resprout** | **resprouts** | **not serotinous** | **serotinous** | **canopy** | **canopy and soil** | **none** | **soil** | **annual** | **biennial** | **perennial** |
| Abundance | Number of species present | Not Rare | 25 | 5 | 1 | 92 | 30 | 3 | 7 | 1 | 0 | 56 | 4 | 0 | 115 |
|  |  | Rare | 5 | 1 | 0 | 17 | 4 | 0 | 2 | 0 | 1 | 10 | 2 | 1 | 20 |
|  | Proportion of species with trait present | Not Rare | 0.833 | 0.833 | 1 | 0.844 | 0.882 | 1 | 0.778 | 1 | 0 | 0.848 | 0.667 | 0 | 0.852 |
|  |  | Rare | 0.167 | 0.167 | 0 | 0.156 | 0.118 | 0 | 0.222 | 0 | 1 | 0.152 | 0.333 | 1 | 0.148 |
| Distribution | Number of species present | Not Rare | 23 | 3 | 1 | 82 | 29 | 3 | 8 | 0 | 0 | 53 | 3 | 0 | 100 |
|  |  | Rare | 7 | 3 | 0 | 27 | 5 | 0 | 1 | 1 | 1 | 13 | 3 | 1 | 35 |
|  | Proportion of species with trait present | Not Rare | 0.767 | 0.5 | 1 | 0.752 | 0.853 | 1 | 0.889 | 0 | 0 | 0.803 | 0.5 | 0 | 0.741 |
|  |  | Rare | 0.233 | 0.5 | 0 | 0.248 | 0.147 | 0 | 0.111 | 1 | 1 | 0.197 | 0.5 | 1 | 0.259 |
| Rabinowitz | Number of species present | Not Rare | 23 | 3 | 1 | 62 | 23 | 3 | 8 | 0 | 0 | 49 | 3 | 0 | 80 |
|  |  | Rare (NSS) | 7 | 3 | 0 | 47 | 11 | 0 | 1 | 1 | 1 | 17 | 3 | 1 | 55 |
|  | Proportion of species with trait present | Not Rare | 0.767 | 0.5 | 1 | 0.569 | 0.676 | 1 | 0.889 | 0 | 0 | 0.742 | 0.5 | 0 | 0.593 |
|  |  | Rare (NSS) | 0.233 | 0.5 | 0 | 0.431 | 0.324 | 0 | 0.111 | 1 | 1 | 0.258 | 0.5 | 1 | 0.407 |

**Table 4**. Number and proportion of rare and not rare species within each trait category in the heath vegetation community.

| Rarity Measure | Number and proportion of species present | Rarity | Fire response trait | | | | Serotiny | | Seed storage location | | | Life history | |
| --- | --- | --- | --- | --- | --- | --- | --- | --- | --- | --- | --- | --- | --- |
|  |  |  | **fire killed** | **fire killed resprouts** | **not fire killed,does not resprout** | **resprouts** | **not serotinous** | **serotinous** | **canopy** | **canopy and soil** | **soil** | **annual** | **perennial** |
| Abundance | Number of species present | Not Rare | 41 | 13 | 1 | 109 | 26 | 9 | 18 | 1 | 75 | 2 | 151 |
|  |  | Rare | 4 | 2 | 0 | 14 | 2 | 1 | 2 | 0 | 6 | 2 | 18 |
|  | Proportion of species with trait present | Not Rare | 0.911 | 0.867 | 1 | 0.886 | 0.929 | 0.9 | 0.9 | 1 | 0.926 | 0.5 | 0.893 |
|  |  | Rare | 0.089 | 0.133 | 0 | 0.114 | 0.071 | 0.1 | 0.1 | 0 | 0.074 | 0.5 | 0.107 |
| Distribution | Number of species present | Not Rare | 36 | 10 | 1 | 95 | 25 | 10 | 19 | 0 | 66 | 1 | 131 |
|  |  | Rare | 9 | 5 | 0 | 28 | 3 | 0 | 1 | 1 | 15 | 3 | 38 |
|  | Proportion of species with trait present | Not Rare | 0.8 | 0.667 | 1 | 0.772 | 0.893 | 1 | 0.95 | 0 | 0.815 | 0.25 | 0.775 |
|  |  | Rare | 0.2 | 0.333 | 0 | 0.228 | 0.107 | 0 | 0.05 | 1 | 0.185 | 0.75 | 0.225 |
| Rabinowitz | Number of species present | Not Rare | 30 | 8 | 1 | 88 | 22 | 9 | 14 | 0 | 56 | 2 | 114 |
|  |  | Rare (NSS) | 15 | 7 | 0 | 35 | 6 | 1 | 6 | 1 | 25 | 2 | 55 |
|  | Proportion of species with trait present | Not Rare | 0.667 | 0.533 | 1 | 0.715 | 0.786 | 0.9 | 0.7 | 0 | 0.691 | 0.5 | 0.675 |
|  |  | Rare (NSS) | 0.333 | 0.467 | 0 | 0.285 | 0.214 | 0.1 | 0.3 | 1 | 0.309 | 0.5 | 0.325 |

**References**

1. Penman, T. D., Binns, D. L., Brassil, T. E., Shiels, R. J. & Allen, R. M. Long-term changes in understorey vegetation in the absence of wildfire in south-east dry sclerophyll forests. *Aust. J. Bot.* **57**, 533–540 (2010).

2. Santana, V. M., Alday, J. G. & Baeza, M. J. Effects of fire regime shift in Mediterranean Basin ecosystems: changes in soil seed bank composition among functional types. *Plant Ecol.* **215**, 555–566 (2014).

3. Watson, P. & Wardell‐Johnson, G. Fire frequency and time-since-fire effects on the open-forest and woodland flora of Girraween National Park, south-east Queensland, Australia. *Austral Ecol.* **29**, 225–236 (2004).

4. Enright, N. J., Marsula, R., Lamont, B. B. & Wissel, C. The ecological significance of canopy seed storage in fire-prone environments: a model for resprouting shrubs. *J. Ecol.* **86**, 960–973 (1998).

5. Foster, C. N. *et al.* Effects of fire regime on plant species richness and composition differ among forest, woodland and heath vegetation. *Appl. Veg. Sci.* **21**, 132–143 (2018).

6. Tozer, M. G. & Bradstock, R. A. Fire-mediated effects of overstorey on plant species diversity and abundance in an eastern Australian heath. *Plant Ecol.* **164**, 213–223 (2003).

7. Veen, G. F. (Ciska), Blair, J. M., Smith, M. D. & Collins, S. L. Influence of grazing and fire frequency on small-scale plant community structure and resource variability in native tallgrass prairie. *Oikos* **117**, 859–866 (2008).

8. Heisler, J. L., Briggs, J. M. & Knapp, A. K. Long-term patterns of shrub expansion in a C4-dominated grassland: fire frequency and the dynamics of shrub cover and abundance. *Am. J. Bot.* **90**, 423–428 (2003).

9. Briggs, J. M., Knapp, A. K. & Brock, B. L. Expansion of Woody Plants in Tallgrass Prairie: A Fifteen-year Study of Fire and Fire-grazing Interactions. *Am. Midl. Nat.* **147**, 287–294 (2002).

10. Bradstock, R. A., Tozer, M. G. & Keith, D. A. Effects of high frequency fire on floristic composition and abundance in a fire-prone heathland near Sydney. *Aust. J. Bot.* **45**, 641–655 (1997).

11. Tessler, N., Sapir, Y., Wittenberg, L. & Greenbaum, N. Recovery of Mediterranean vegetation after recurrent forest fires: insight from the 2010 forest fire on Mount Carmel, Israel. *Land Degrad. Dev.* **27**, 1424–1431 (2016).

12. Pausas, J. G. & Bradstock, R. A. Fire persistence traits of plants along a productivity and disturbance gradient in mediterranean shrublands of south-east Australia. *Glob. Ecol. Biogeogr.* **16**, 330–340 (2007).

13. Bradstock, R. A. & O’connell, M. A. Demography of woody plants in relation to fire: Banksia ericifolia L.f. and Petrophile pulchella (Schrad) R.Br. *Aust. J. Ecol.* **13**, 505–518 (1988).

14. Ross, K. A., Fox, B. J. & Fox, M. D. Changes to plant species richness in forest fragments: fragment age, disturbance and fire history may be as important as area. *J. Biogeogr.* **29**, 749–765 (2002).

15. Russell, R. P. & Parsons, R. F. Effects of Time Since Fire on Heath Floristics at Wilson’s Promontory, Southern Australia. *Aust. J. Bot.* **26**, 53–61 (1978).

16. Hobbs, R. J. & Atkins, L. Fire-Related Dynamics of a Banksia Woodland in South-Western Western Australia. *Aust. J. Bot.* **38**, 97–110 (1990).

17. Duff, T. J., Bell, T. L. & York, A. Managing multiple species or communities? Considering variation in plant species abundances in response to fire interval, frequency and time since fire in a heathy Eucalyptus woodland. *For. Ecol. Manag.* **289**, 393–403 (2013).

18. Williams, P. R., Congdon, R. A., Grice, A. C. & Clarke, P. J. Effect of fire regime on plant abundance in a tropical eucalypt savanna of north‐eastern Australia. *Austral Ecol.* **28**, 327–338 (2003).

19. Gosper, C. R., Yates, C. J., Prober, S. M. & Parsons, B. C. Contrasting changes in vegetation structure and diversity with time since fire in two Australian Mediterranean-climate plant communities. *Austral Ecol.* **37**, 164–174 (2012).

20. Wright, B. R. & Clarke, P. J. Fire regime (recency, interval and season) changes the composition of spinifex (Triodia spp.)-dominated desert dunes. *Aust. J. Bot.* **55**, 709–724 (2007).

21. Morrison, D. A. *et al.* Effects of fire frequency on plant species composition of sandstone communities in the Sydney region: Inter‐fire interval and time‐since‐fire. *Aust. J. Ecol.* **20**, 239–247 (1995).

22. Morrison, D. A. & Renwick, J. A. Effects of variation in fire intensity on regeneration of co-occurring species of small trees in the Sydney region. *Aust. J. Bot.* **48**, 71–79 (2000).

23. Delitti, W., Ferran, A., Trabaud, L. & Vallejo, V. R. Effects of fire recurrence in Quercus coccifera L. shrublands of the Valencia Region (Spain): I. plant composition and productivity. *Plant Ecol.* **177**, 57–70 (2005).

24. Penman, T. D., Binns, D. L., Shiels, R. J., Allen, R. M. & Kavanagh, R. P. Changes in understorey plant species richness following logging and prescribed burning in shrubby dry sclerophyll forests of south‐eastern Australia. *Austral Ecol.* **33**, 197–210 (2008).

25. Close, D. C. *et al.* Premature Decline of Eucalyptus and Altered Ecosystem Processes in the Absence of Fire in Some Australian Forests. *Bot. Rev.* **75**, 191–202 (2009).

26. Lunt, I. D. Allocasuarina (Casuarinaceae) Invasion of an Unburnt Coastal Woodland at Ocean Grove, Victoria: Structural Changes 1971–1996. *Aust. J. Bot.* **46**, 649–656 (1998).

27. Australian Bureau of Meteorology. Climate Data Online. www.bom.gov.au (2019).

28. Keith, D. A. *Ocean shores to desert dunes: the native vegetation of NSW and the ACT (Selected Extracts)*. (Department of Environment and Conservation (NSW), 2004).

29. Taws, N. *Vegetation survey and mapping of Jervis Bay Territory*. (Taws Botanical Research, 1997).

30. Gaston, K. J. What is rarity? in *Rarity* 1–21 (Springer, 1994).

31. Rabinowitz, D. Seven forms of rarity. in *The biological aspects of rare plant conservation* (ed. Synge, H.) 205–217 (John Wiley and Sons: Chichester, UK, 1981).

32. Lenth, R., Singmann, H., Love, J. & Buerkner, P. M., Herve. emmeans: Estimated Marginal Means, aka Least-Squares Means. (2020).
